# Supplementary material for: Br−/BrO−-mediated highly efficient photoelectrochemical epoxidation of alkenes on α-Fe2O3
Source: Nat Commun. 2023 Apr 7;14:1943. doi: 10.1038/s41467-023-37620-8 (PMC10082182; doi:10.1038/s41467-023-37620-8)
Supplement: Supplementary file 1 — Supplementary Information [file 41467_2023_37620_MOESM1_ESM.pdf]

Br^-/BrO^--mediated Highly Efficient Photoelectrochemical Epoxidation of Alkenes on α-Fe_2O_3

Yukun Zhao, Mengyu Duan, Chaoyuan Deng, Jie Yang, Sipeng Yang, Yuchao Zhang, Hua Sheng, Youji Li, Chuncheng Chen*, and Jincai Zhao

Energy balance

An estimation of the energy balance for the pure electrochemical polarization vers the photoelectrochemical route on α-Fe_2O_3 was conducted.

Energy balance (E_b) = Energy Input (E_i) - Energy Output (E_o)

In this system, energy output refers to the energy consumption from alkene (**1**) to epoxide (**2**). Therefore, the values of energy output should be a constant (1 mol of **2**), regardless of electrochemical or photoelectrochemical systems. In photoelectrochemical system, the energy input is composed with electric input (E_{ie}) and light input (E_{is}). In these systems, the cost of energy input is determined by the electric consumption, because the cost of light input could be from the solar energy, which is cheap and "green". The electric consumption (E_{ie}) can be estimated by:

$$\mathbf{E_{ie} = n \times m \times F \times U / \eta_{FE}}$$

Where **n** is the number of electrons (2); **m** is the mole of epoxide (mol); **F** is faradaic constant (96485 C/mol); **U** is applied bias (V); η_{FE} is the Faradic efficiency (82%)

The energy difference between the EC and PEC systems is determined by difference in the applied bias (Δ**U**):

$$\mathbf{\Delta E_{ie} = n \times m \times F \times \Delta U / \eta_{FE}} = 211.8 \text{ kJ/mol}$$

At 0.6 mA/cm^2, the difference in the applied bias between EC and PEC systems is Δ**U** = 0.90 V. The energy difference is 211.8 kJ/mol, which should be supplied by the light energy.

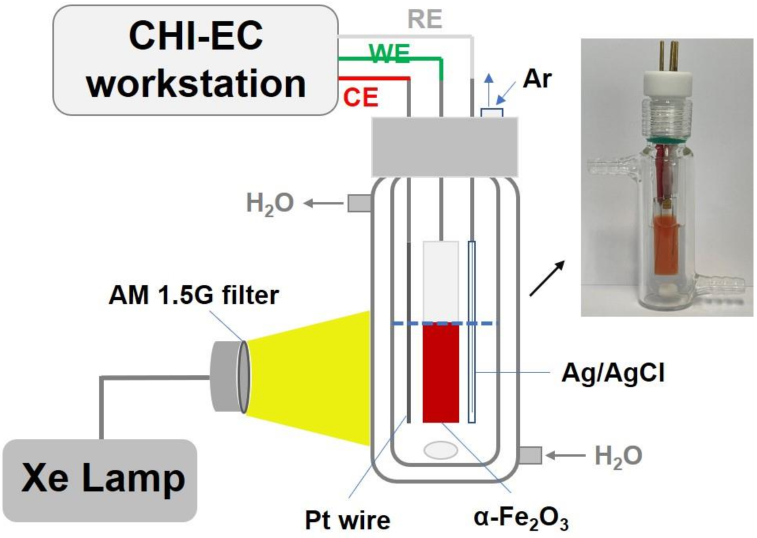

Supplementary Fig. 1 PEC setup. In the 10-ml PEC cell, the effective geometric surface area of the hematite photoanode was 1×2 cm^2, and the volume was 5 ml with CH_3CN (5 % H_2O) solution. In the PEC epoxidation reactions, the applied bias was 0.15 V vs. Fc/Fc^+ with 10 mM substrates and 100 mM TBABr in the Ar atmosphere at 25 ^oC.

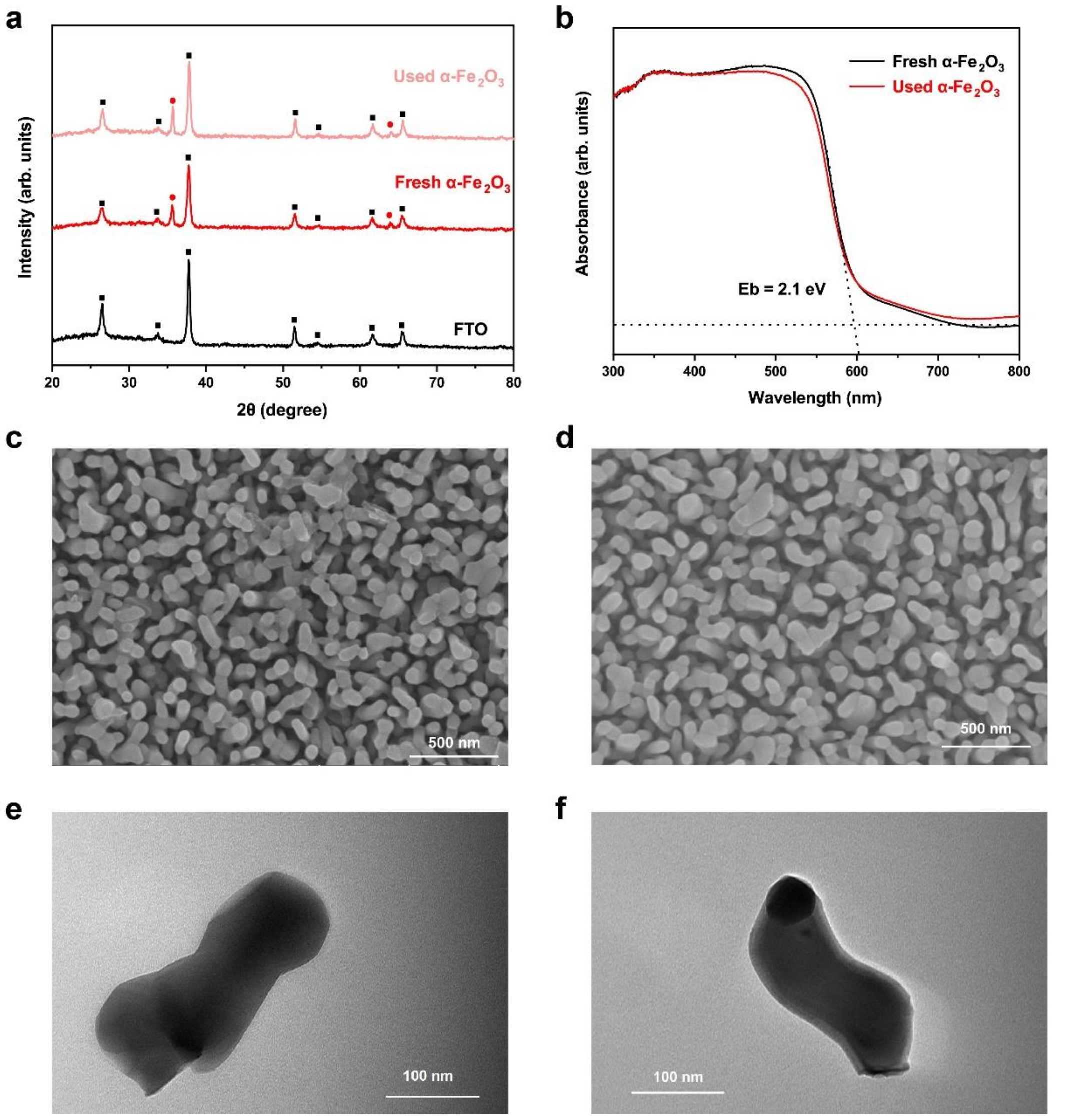

Supplementary Fig. 2 The characterizations of fresh and used α-Fe_2O_3 photoanodes.

(**a**) XRD spectra; (**b**) UV-vis diffuse spectra; SEM images of fresh (**c**) and used (**d**) α-Fe_2O_3; TEM images of fresh (**e**) and used (**f**) α-Fe_2O_3.

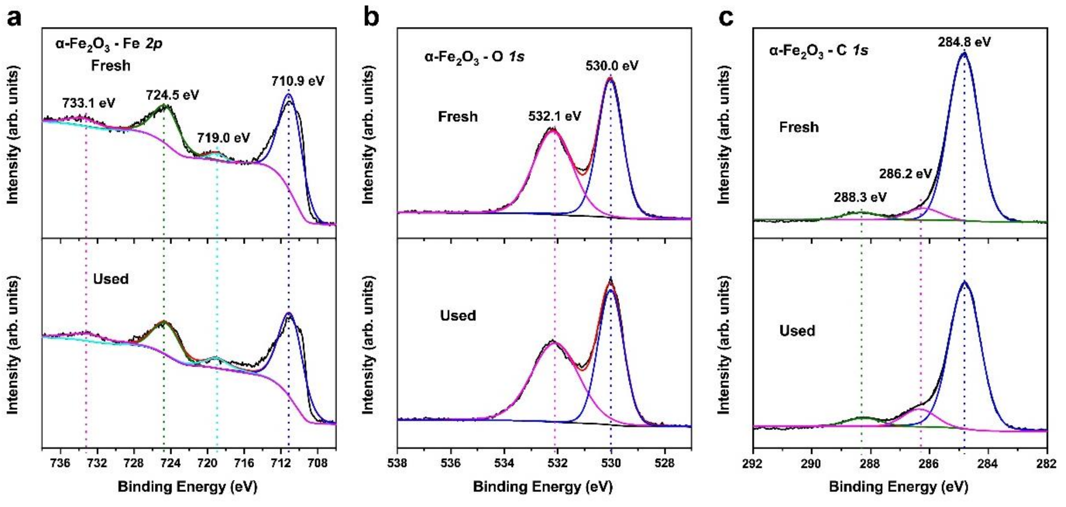

Supplementary Fig. 3 The XPS spectra of fresh and used α-Fe_2O_3 photoanodes. (**a**) XPS spectra of Fe *2p* core level; (**b**) XPS spectra of O *1s* core level; and (**c**) XPS spectra of C *1s* core level.

As shown in Supplementary Fig. 2a, the XRD peaks located at 35.6° and 64.0° are indexed to the (110) and (300) lattice diffractions of α-Fe_2O_3 (JCPDS: 33-0664), in which the red circles and black squares represent the diffraction peaks of hematite and FTO, respectively. For UV-vis diffuse spectrum (Supplementary Fig. 2b), the maximum light absorption wavelength of α-Fe_2O_3 can reach to ~600 nm, indicating that the band gap of α-Fe_2O_3 is about 2.1 eV. According to SEM and TEM (Supplementary Figs. 2c and 2e), the α-Fe_2O_3 film consists of nanorods with a diameter size in the range of 50-100 nm, which grew perpendicular to the FTO surface. For XPS analysis of pristine α-Fe_2O_3 (Supplementary Fig. 3), the peaks located at 710.9 and 724.5 eV are assigned to Fe $2p_{3/2}$ and Fe $2p_{1/2}$[1]. The satellite peak of Fe^{3+} at 719.0 eV is observed, but no any Fe^{2+} satellite peak is appeared[2]. For O *1s* XPS spectrum, the strong peak at approximately 530.0 eV is attributed to the lattice oxygen species of pristine α-Fe_2O_3, while the shoulder peak centered at 532.1 eV is assigned to the surface labile oxygen species (such as surface hydroxyls)[3].

After suffering from PEC reaction, the crystalline phase (XRD), the light absorption (UV-vis), the morphography (SEM and TEM) and surface components (including valence states, XPS) have not been changed obviously, indicative of that α-Fe_2O_3 is stable in the PEC Br-mediated epoxidation reaction, which is consistent with the result of repeated experiments (Supplementary Fig. 11).

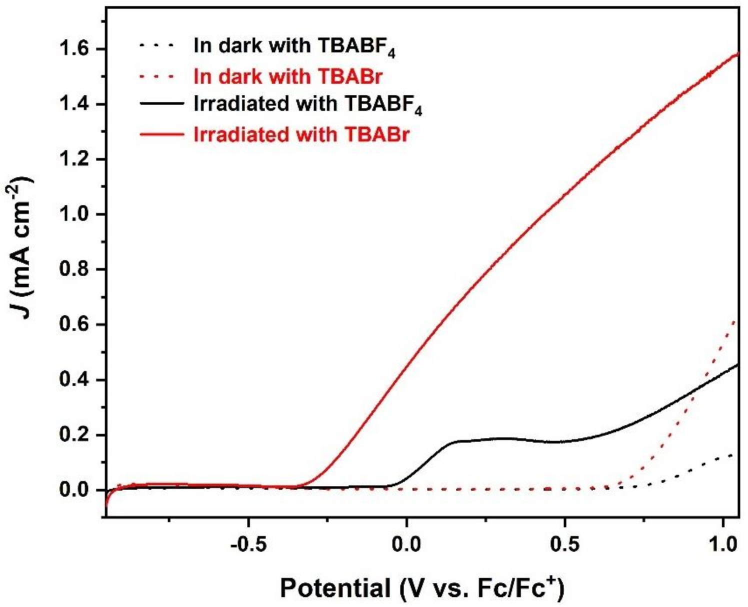

Supplementary Fig. 4 *J*–*V* scan of α-Fe_2O_3 measured in 0.1 M $TBABF_4$ (black lines) and TBABr (red lines) solution (CH_3CN with 5% H_2O) under an Ar atmosphere under AM 1.5G illumination (solid) and in the dark condition (dash). Even in the dark conditions, the onset potential in the TBABr system is still superior to that in the $TBABF_4$ system.

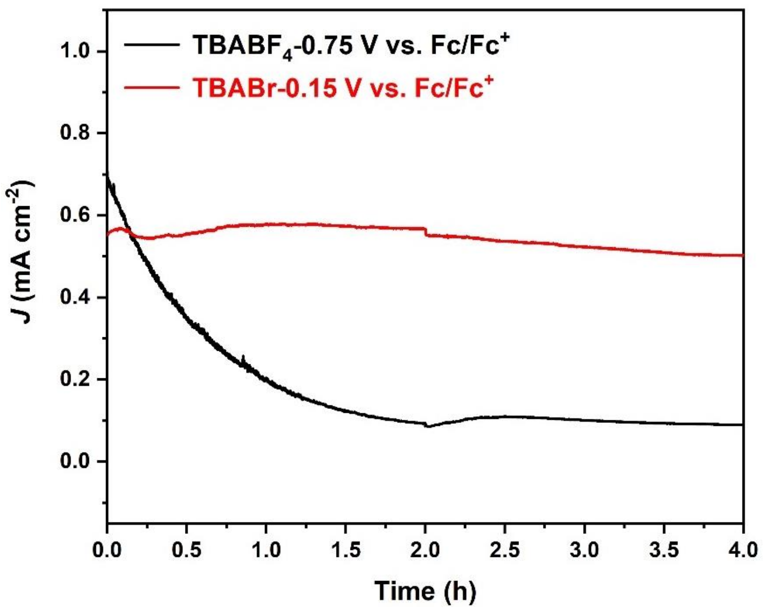

Supplementary Fig. 5 Chronoamperometry curves during the PEC oxidation of substrate **1** on α-Fe_2O_3 with $TBABF_4$ at 0.75 V vs. Fc/Fc^+ and TBABr at 0.15 V vs. Fc/Fc^+.

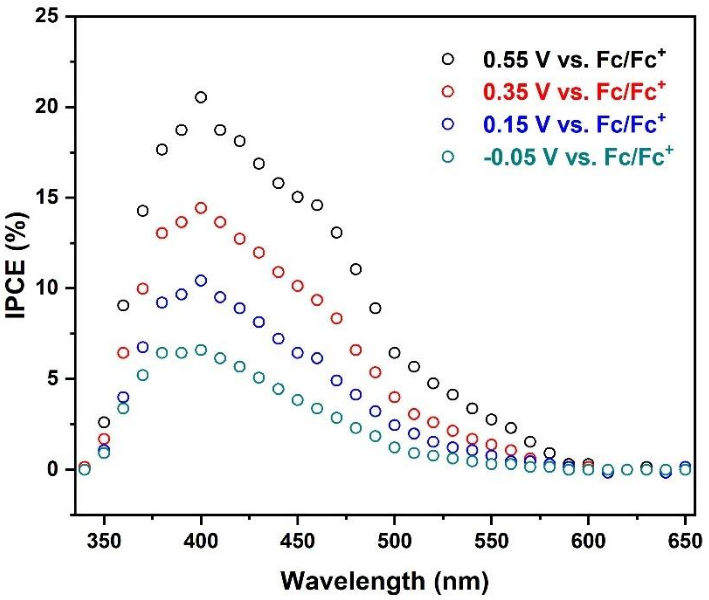

Supplementary Fig. 6 The monochromatic incident photon-to-electron conversion efficiency (IPCE) on α-Fe_2O_3 with different applied biases in Br-mediated epoxidation system. It is found that the IPCE increases with the shortened wavelength from ~600 nm, and exhibits the maximum values at the wavelength of 400 nm, where the IPCE value is ~21% at 0.55 V vs. Fc/Fc^+.

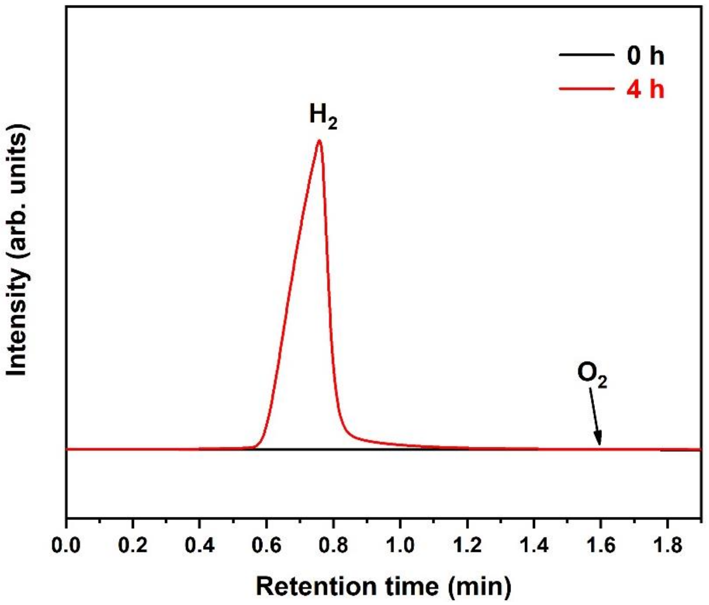

Supplementary Fig. 7 The GC spectra of the headspace gas of the PEC cell after 4 hours' photoelectrolysis. The Faradaic Efficiency of the hydrogen evolution was 90%. No dioxygen (if formed, its retention time should be at ~ 1.6 min) was detected in GC spectra, which indicates that water oxidation to O_2 rarely occurs in the Br-mediated PEC epoxidation.

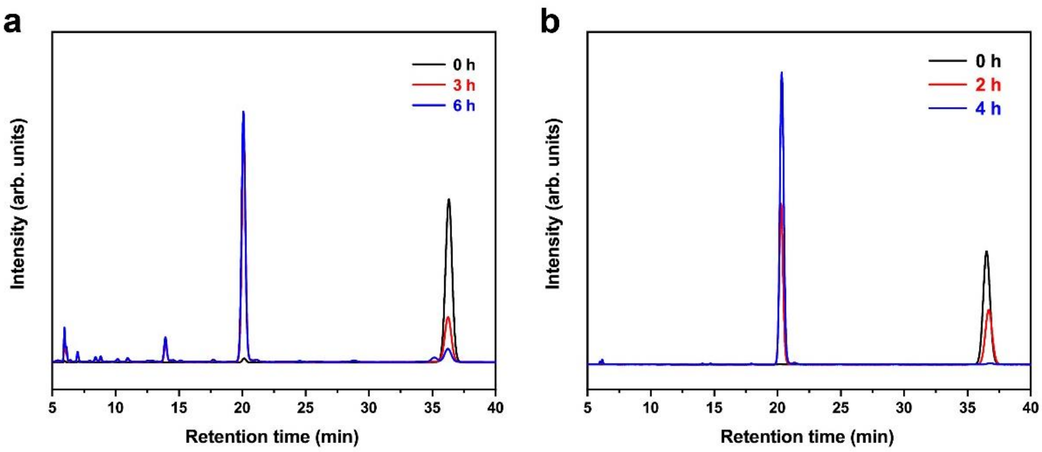

Supplementary Fig. 8 The HPLC spectra obtained at different PEC oxidation time of 10 mM **1** with (**a**) 100 mM $TBABF_4$ and 3 mM $NaBrO_3$ at 0.75 V vs. Fc/Fc^+ and (**b**) 20 mM NaBr at 0.35 V vs. Fc/Fc^+ on α-Fe_2O_3.

In the presence of 3 mM BrO_3^-, the selectivity and FE of epoxide is 64% and 41%, respectively, which is similar to that without BrO_3^- (in pure $TBABF_4$ system, selectivity 43±5%, FE 41±3%, respectively), indicating that the BrO_3^- is not active toward epoxidation reaction. By contrast, by using 20 mM NaBr to substitute TBABr, an excellent selectivity (> 95%) of epoxide was achieved. These results confirmed that Br^- serves as a key mediator to effectively perform alkene epoxidation.

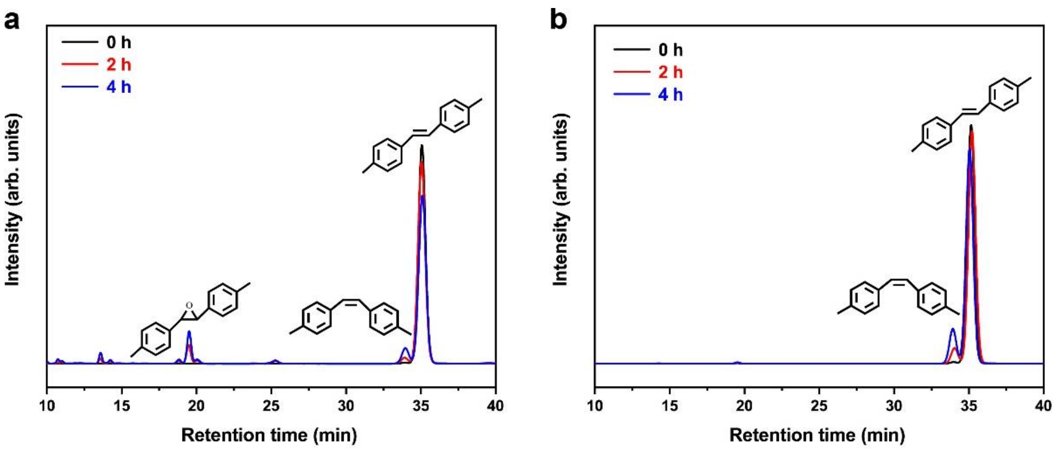

Supplementary Fig. 9 The HPLC spectra obtained at different PEC oxidation time of 10 mM **1** with 100 mM (**a**) TBACl or (**b**) TBAI at 0.15 V vs. Fc/Fc^+ on α-Fe_2O_3.

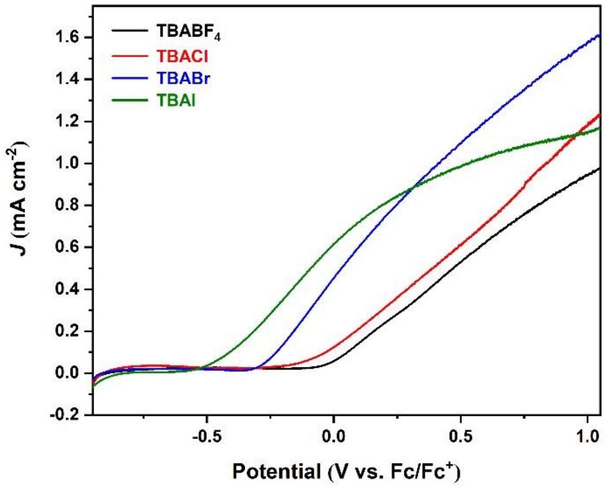

Supplementary Fig. 10 *J–V* scan of α-Fe_2O_3 with different 100 mM TBAX (X^-: BF_4^-, Cl^-, Br^- and I^-) under AM 1.5G illumination measured in CH_3CN solution with 5% H_2O in an Ar atmosphere with 10 mM **1**. Scan rate 0.05 V s^{-1}.

Contrary to Br^--mediator, epoxidation reactions by using both Cl^- and I^- as the mediator exhibit the poor activity and selectivity. For the TBACl system, the epoxide is detected with poor performance, while in the TBAI system no oxidation of alkene is observed (Supplementary Fig. 9). The LSV experiments show that, in the system using TBACl as electrolyte, the negative shift of onset potential and the photocurrent increase relative to the case of $TBABF_4$ are not very significant (Supplementary Fig. 10), which suggests that the oxidation of Cl^- is not very competitive to the water oxidation on α-Fe_2O_3 photoanode under our experimental conditions. Therefore, poor epoxidation performance of the TBACl systems should originate from the unfavorable oxidation of Cl^- to ClO^-. For the TBAI system, the large negative shift of onset potential indicates that I^- is easy to be oxidized on α-Fe_2O_3 photoanode. The low epoxidation activity may be attributed to the low oxidation ability and the poor stability of IO^- in the TBAI system. For TBABr system, however, the Br^- is facile to be oxygenated to BrO^- species on α-Fe_2O_3 under our PEC conditions, and the formed BrO^- species is active enough to transfer its oxygen atom to the alkenes. Therefore, the Br^-/BrO^- cycling plays an unique role in mediating the epoxidation.

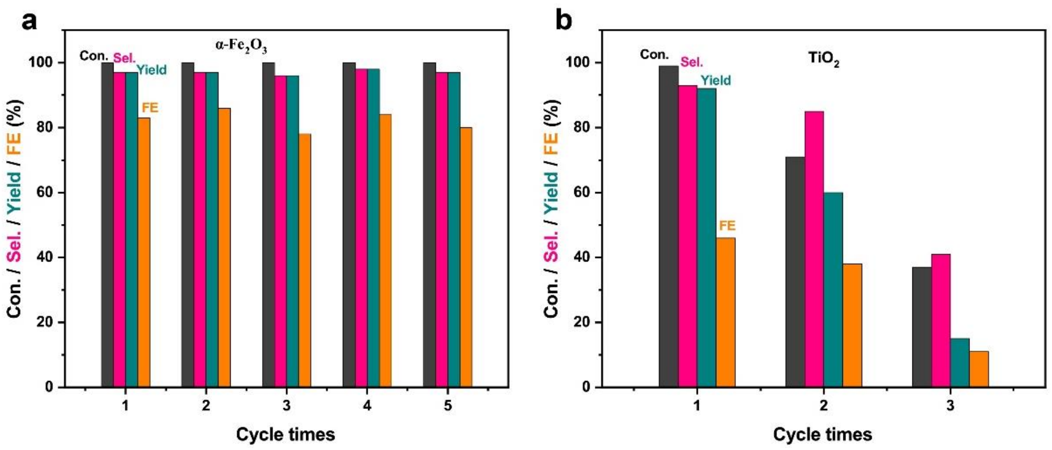

Supplementary Fig. 11 The repeated experiments for the Br-mediated epoxidation of substrate **1** by using the same photoanodes of α-Fe_2O_3 (**a**) and TiO_2 (**b**).

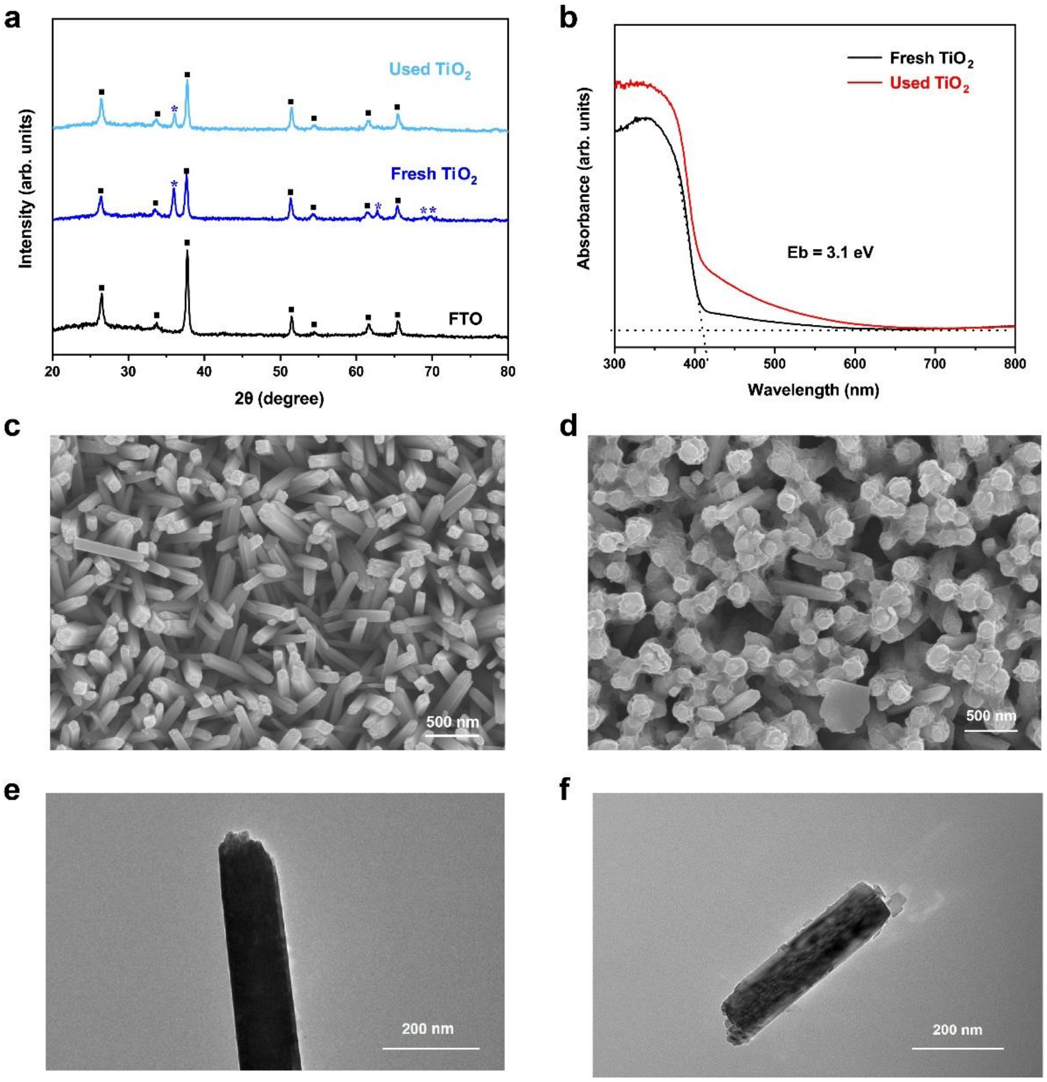

Supplementary Fig. 12 The characterizations of fresh and used TiO_2 photoanodes. (**a**) XRD spectra; (**b**) UV-vis diffuse spectra; SEM images of fresh (**c**) and used (**d**) TiO_2; TEM images of fresh (**e**) and used (**f**) TiO_2.

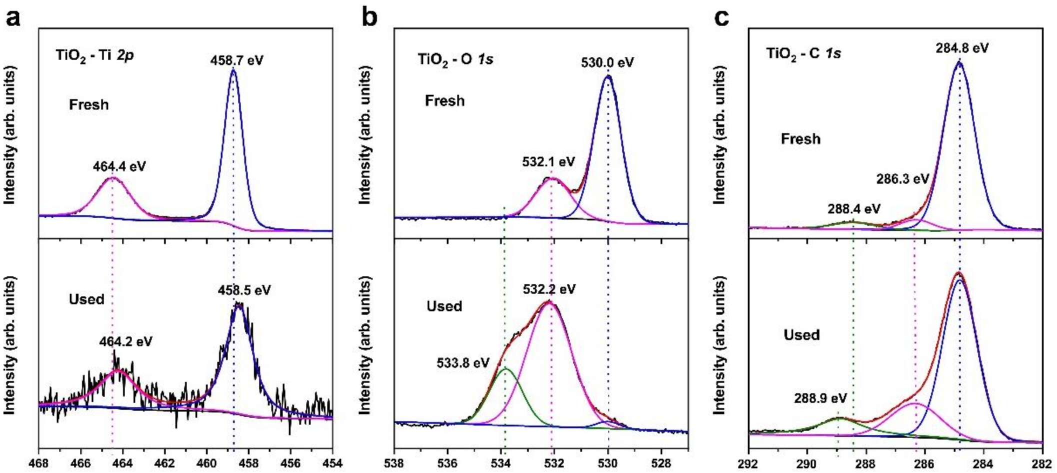

Supplementary Fig. 13 The XPS spectra of fresh and used α-Fe_2O_3 photoanodes. (**a**) XPS spectra of Ti *2p* core level; (**b**) XPS spectra of O *1s* core level; and (**c**) XPS spectra of C *1s* core level.

For TiO_2, as shown in Supplementary Fig. 12a, the XRD peaks located at 36.1°, 62.7°, 69.0° and 69.8° (blued asterisk) are indexed to the (101), (002), (301) and (112) lattice diffractions of rutile TiO_2 (JCPDS: 21-1276). For UV-vis diffuse spectrum (Supplementary Fig. 12b), the maximum light absorption wavelength of TiO_2 only reaches to ~400 nm, indicating that the band gap is about 3.1 eV. The SEM and TEM images (Supplementary Figs. 12c and 12e) show that the TiO_2 photoanode consists of uniform nanorods with the diameter of around 100 nm. In XPS spectra (Supplementary Fig. 13), the peaks located at 458.7and 464.4 eV are assigned to Ti $2p_{3/2}$ and Ti $2p_{1/2}$, attributing to Ti^{4+} species[4]. Two peaks in O 1s spectrum of TiO_2, which are located at 530.0 and 532.1 eV, are corresponded to lattice O species and surface-active oxygen species, respectively.

Different from α-Fe_2O_3 photoanodes, as shown in Supplementary Fig. 13, TiO_2 is found to be changed significantly after the Br-mediated PEC reaction. Particularly, in the used TiO_2 photoanode, a new absorption in the wavelength range of 400 ~ 650 nm in the UV-vis spectra appears (Supplementary Fig. 12b). More O*1s* and C*1s* peaks in the XPS results are observed (Supplementary Fig. 13). The SEM image shows that the surface of anode seems to be covered by some amorphous species (Supplementary Fig. 12d). All these results suggest that some organic by-products during PEC Br-mediated epoxidation process are formed and deposited on the surface of TiO_2, which is consistent with the poorer selectivity and the rapid decay in the PEC activity of TiO_2 photoanode (Supplementary Fig. 11).

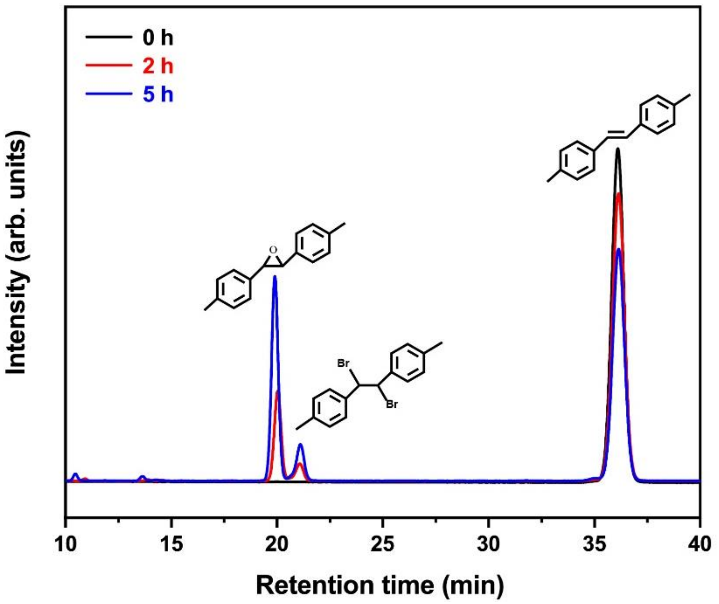

Supplementary Fig. 14 The HPLC spectra of **2** with Pt foil (2 × 0.5 cm^2) as anode. The electrolysis reactions were conducted at 1.05 V vs. Fc/Fc^+.

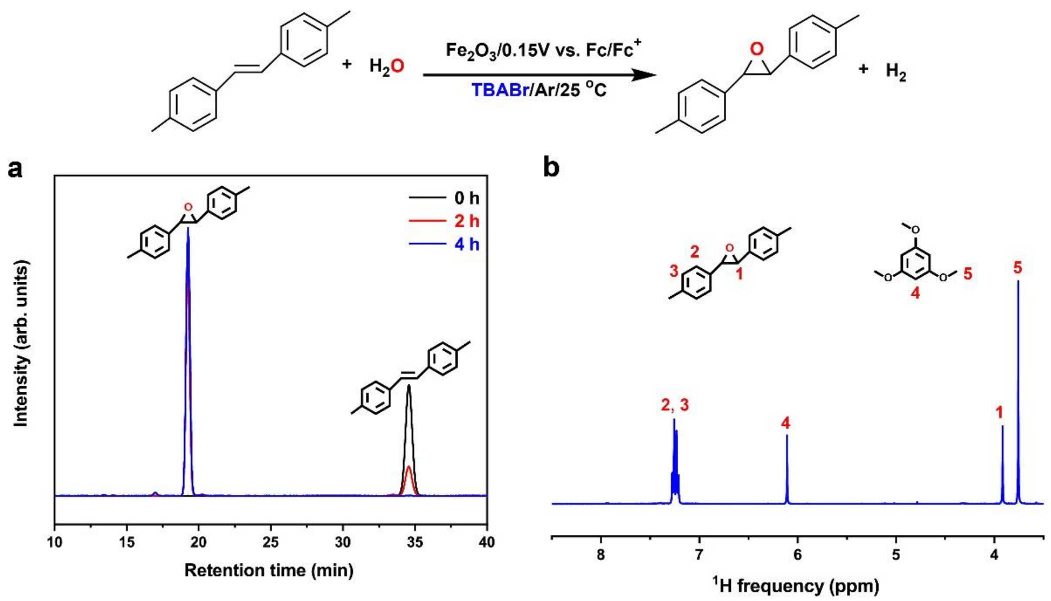

Supplementary Fig. 15 The HPLC spectra (**a**) and the corresponding ^{1}H NMR spectra (**b**) of **2**. The PEC reactions were conducted at 0.15 V vs. Fc/Fc^+ applied bias. An internal standard 1,3,5-trimethoxybenzene (4, 6.11 ppm, s, 3H) was added to quantify the epoxides (1, 3.92 ppm, s, 2H) in CD_3CN. The conversion of **1** at 4 h was 100%, and the corresponding yield of epoxide **2** was 97±1%.

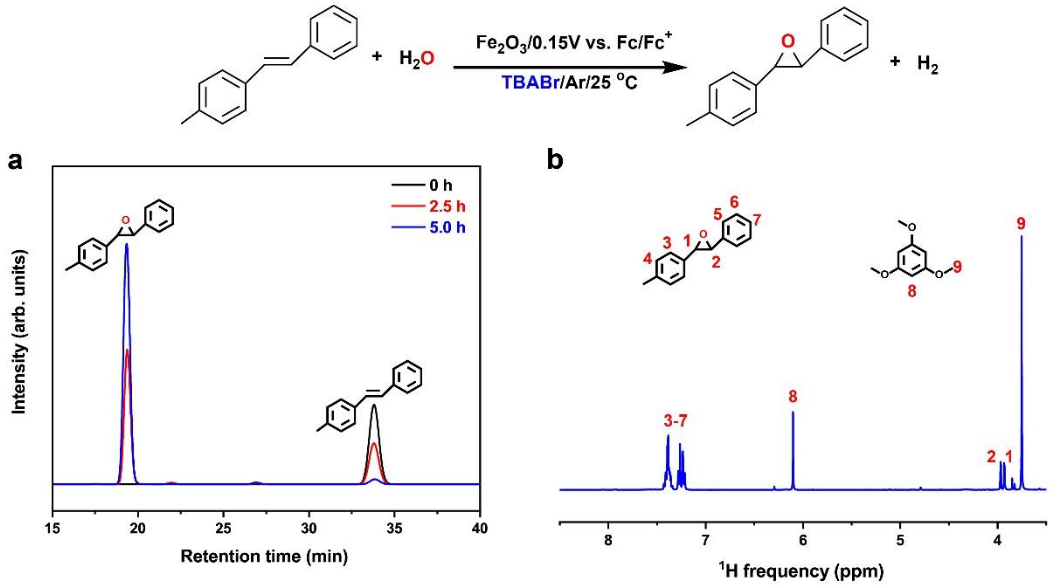

Supplementary Fig. 16 The HPLC spectra (**a**) and the corresponding ^{1}H NMR spectra (**b**) of **3**. The PEC reactions were conducted at 0.15 V vs. Fc/Fc$^+$ for 5 h. An internal standard 1,3,5-trimethoxybenzene (8, 6.11 ppm, s, 3H) was added to quantify the epoxides (1, 3.93 ppm, d, 1H; 2, 3.97 ppm, d, 1H) in CD_3CN. The conversion of 4-CH_3-*trans*-stilbene at 5 h was 90±4%, and the corresponding yield of epoxide **3** was 87±5%.

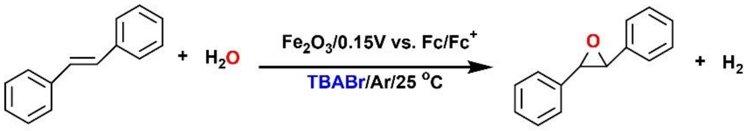

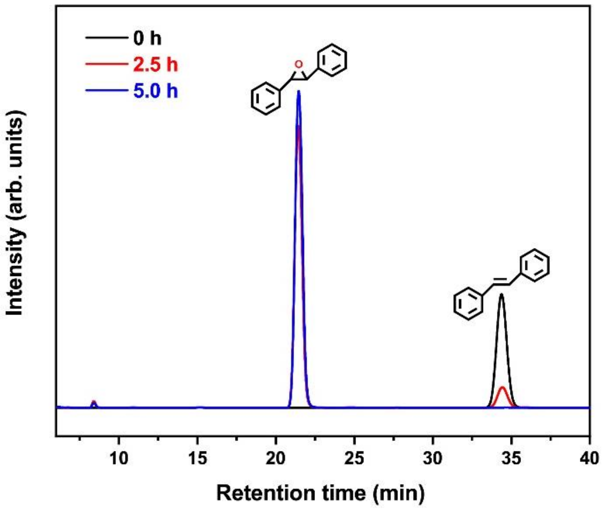

Supplementary Fig. 17 The HPLC spectra of **4**. The PEC reactions were conducted at 0.15 V vs. Fc/Fc^{+} for 5 h. The substrate (*trans*-stilbene) and product **4** were qualified by the standard curves. The conversion of *trans*-stilbene at 5 h was 100%, and the corresponding yield of epoxide **4** was 96±3%.

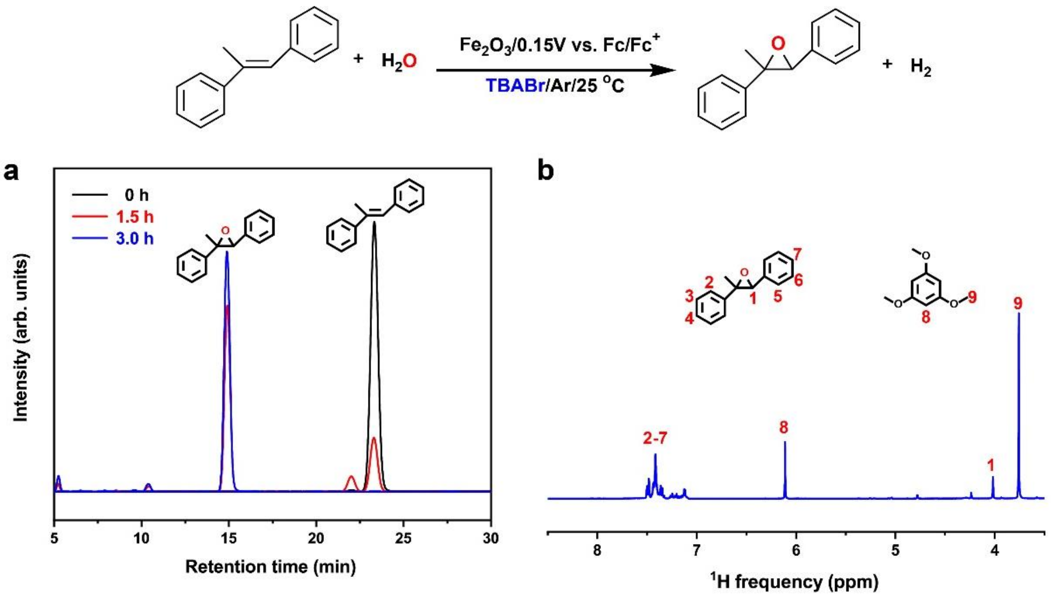

Supplementary Fig. 18 The HPLC spectra (**a**) and the corresponding ^{1}H NMR spectra (**b**) of **5**. The PEC reactions were conducted at 0.15 V vs. Fc/Fc$^+$ for 3 h. An internal standard 1,3,5-trimethoxybenzene (8, 6.11 ppm, s, 3H) was added to quantify the epoxides (1, 4.02 ppm, s, 1H) in CD_3CN. The conversion of *Alfa*-CH_3-*trans*-stilbene at 3 h was 99±1%, and the corresponding yield of epoxide **5** was 92±2%.

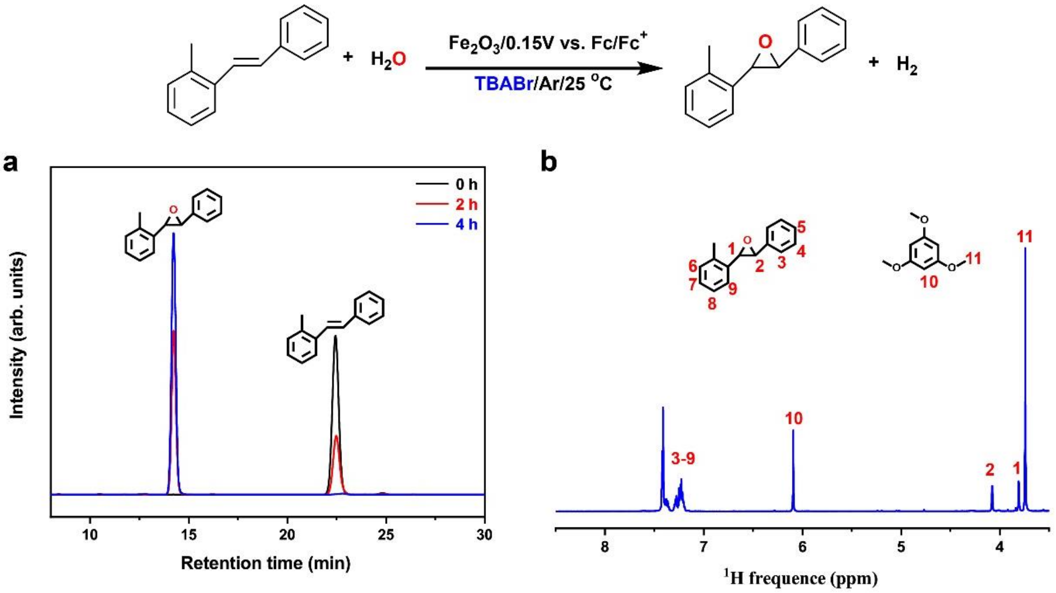

Supplementary Fig. 19 The HPLC spectra (**a**) and the corresponding ^{1}H NMR spectra (**b**) of **6**. The PEC reactions were conducted at 0.15 V vs. Fc/Fc^{+} for 4 h. An internal standard 1,3,5-trimethoxybenzene (10, 6.11 ppm, s, 3H) was added to quantify the epoxides (1, 3.81 ppm, d, 1H; 2, 4.08 ppm, d, 1H) in CD_3CN. The conversion of 2-CH_3-*trans*-stilbene at 4 h was >99%, and the corresponding yield of epoxide **6** was 97±1%.

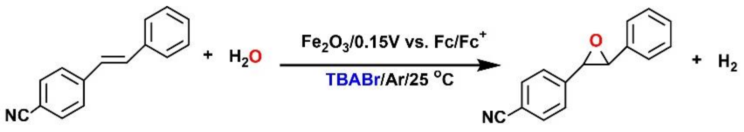

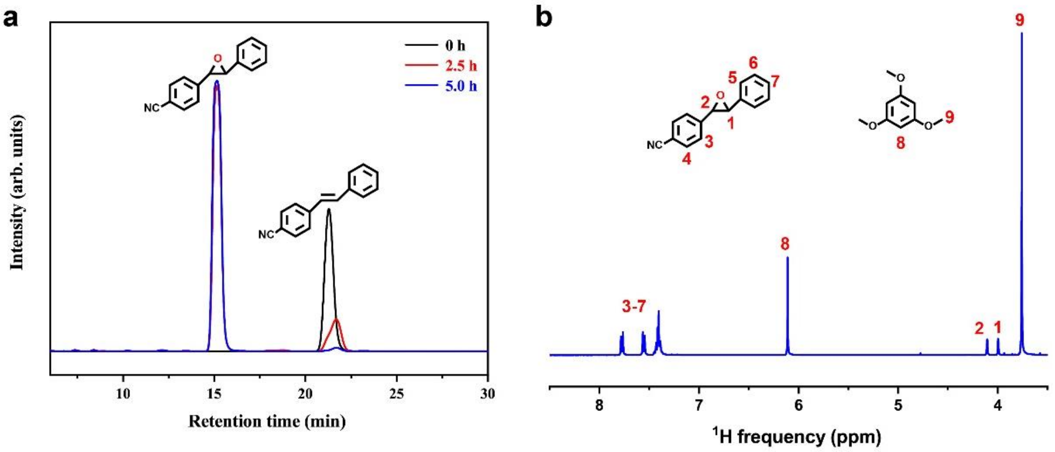

Supplementary Fig. 20 The HPLC spectra (**a**) and the corresponding ^{1}H NMR spectra (**b**) of **7**. The PEC reactions were conducted at 0.15 V vs. Fc/Fc$^+$ for 5 h. An internal standard 1,3,5-trimethoxybenzene (8, 6.11 ppm, s, 3H) was added to quantify the epoxides (1, 3.99 ppm, d, 1H; 2, 4.10 ppm, d, 1H) in CD_3CN. The conversion of 4-CN-*trans*-stilbene at 5 h was 91±6%, and the corresponding yield of epoxide **7** was 89±6%.

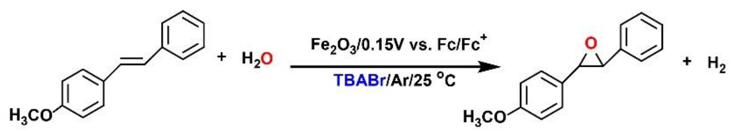

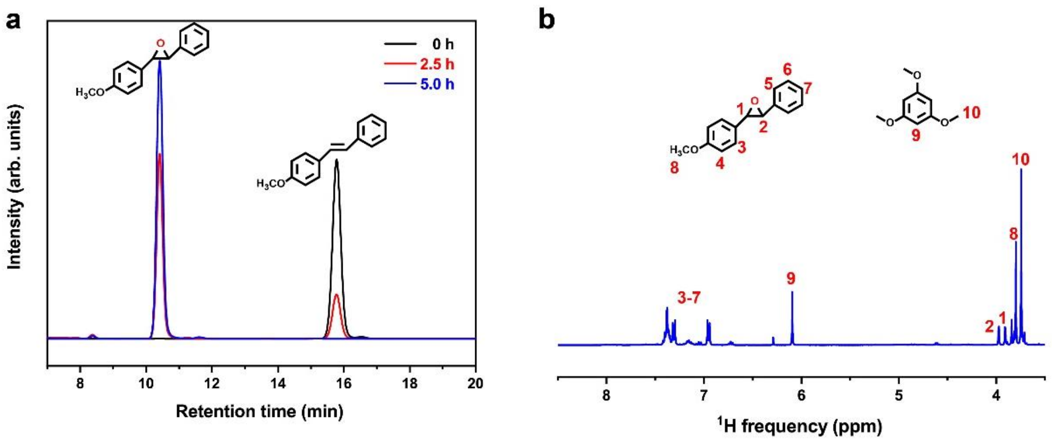

Supplementary Fig. 21 The HPLC spectra (**a**) and the corresponding ^{1}H NMR spectra (**b**) of **8**. The PEC reactions were conducted at 0.15 V vs. Fc/Fc^+ for 5 h. An internal standard 1,3,5-trimethoxybenzene (9, 6.11 ppm, s, 3H) was added to quantify the epoxides (1, 3.91 ppm, d, 1H; 2, 3.97 ppm, d, 1H) in CD_3CN. The conversion of 4-OCH_3-*trans*-stilbene at 5 h was 99±1%, and the corresponding yield of epoxide **8** was 96±2%.

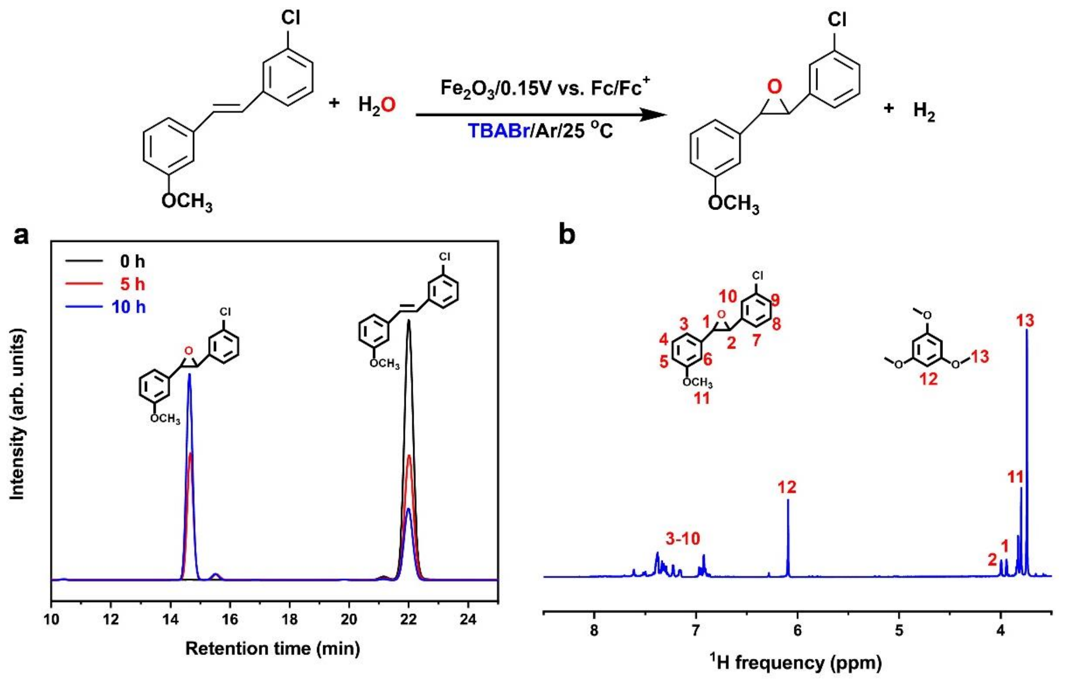

Supplementary Fig. 22 The HPLC spectra (**a**) and the corresponding ^{1}H NMR spectra (**b**) of **9**. The PEC reactions were conducted at 0.15 V vs. Fc/Fc^{+} for 10 h. An internal standard 1,3,5-trimethoxybenzene (12, 6.11 ppm, s, 3H) was added to quantify the epoxides (1, 3.94 ppm, d, 1H; 2, 4.00 ppm, d, 1H) in CD_3CN. The conversion of 3-Cl-3'-OCH_3-*trans*-stilbene at 10 h was 74±1%, and the corresponding yield of epoxide **9** was 73±1%.

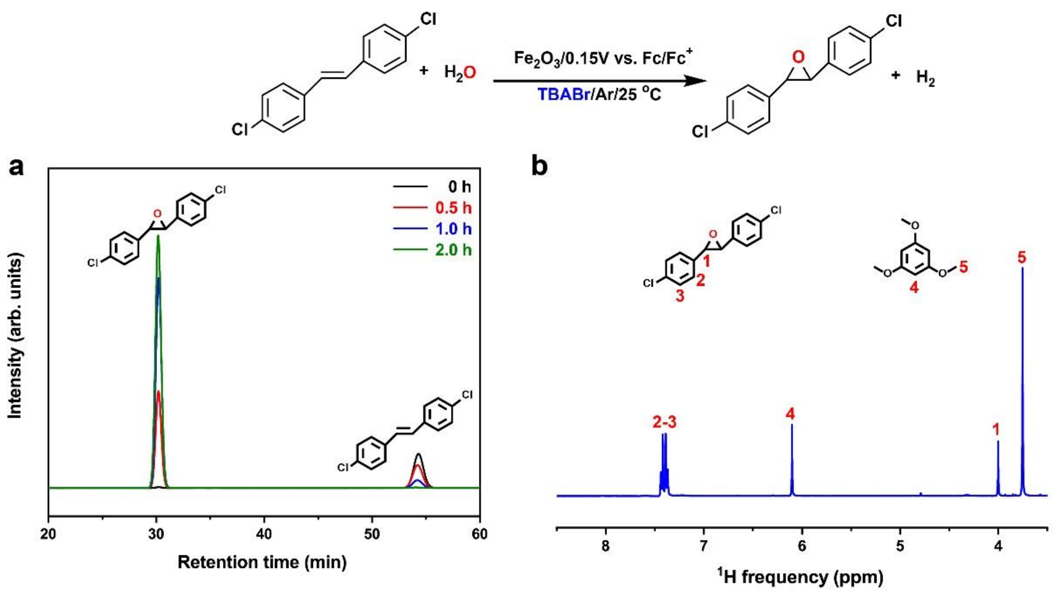

Supplementary Fig. 23 The HPLC spectra (**a**) and the corresponding ^{1}H NMR spectra (**b**) of **10**. The initial concentration of substrate was 5.0 mM. The PEC reactions were conducted at 0.15 V vs. Fc/Fc$^+$ for 2 h. An internal standard 1,3,5-trimethoxybenzene (4, 6.11 ppm, s, 3H) was added to quantify the epoxides (1, 4.00 ppm, s, 2H) in CD_3CN. The conversion of 4,4'-Cl_2-*trans*-stilbene at 2 h was >98%, and the corresponding yield of epoxide **10** was 89±7%.

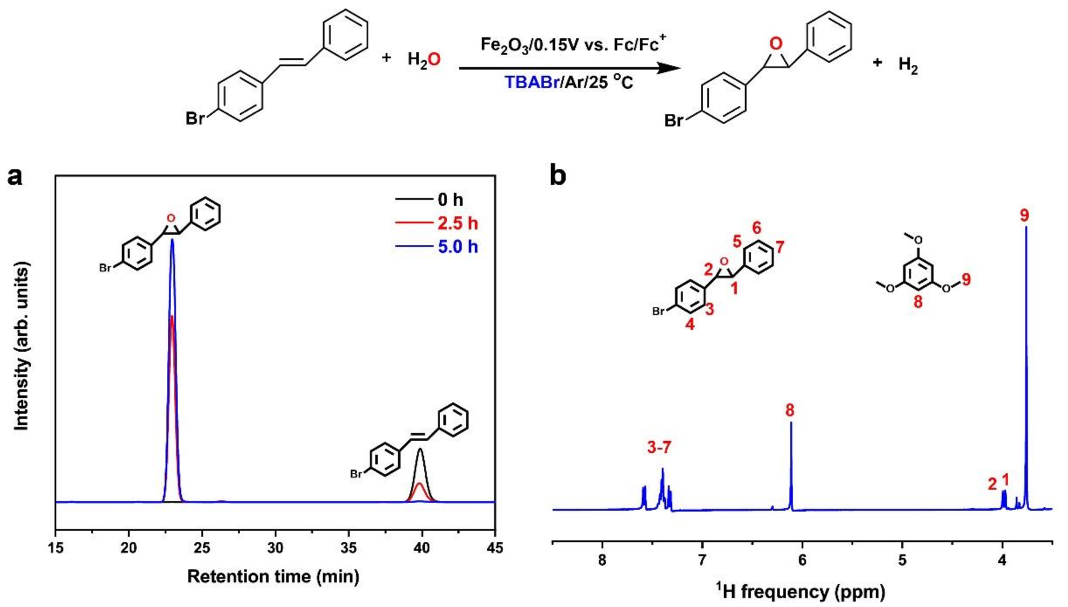

Supplementary Fig. 24 The HPLC spectra (**a**) and the corresponding ^{1}H NMR spectra (**b**) of **11**. The PEC reactions were conducted at 0.15 V vs. Fc/Fc^{+} for 5 h. An internal standard 1,3,5-trimethoxybenzene (8, 6.11 ppm, s, 3H) was added to quantify the epoxides (1, 3.97 ppm, d, 1H; 2, 3.99 ppm, d, 1H) in CD_3CN. The conversion of 4-Br-*trans*-stilbene at 5 h was 96±3%, and the corresponding yield of epoxide **11** was 95±3%.

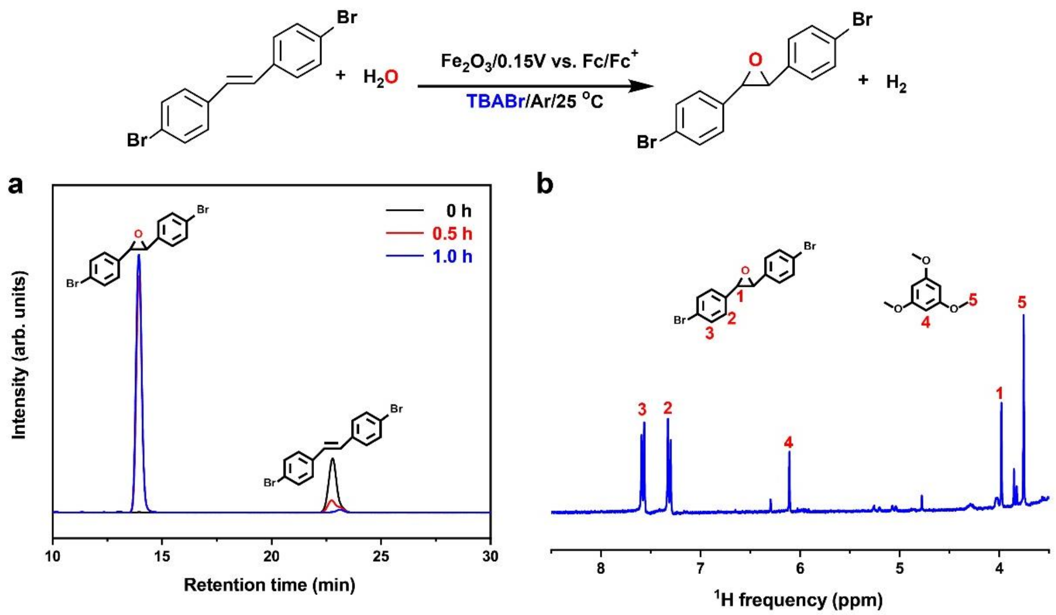

Supplementary Fig. 25 The HPLC spectra (**a**) and the corresponding ^{1}H NMR spectra (**b**) of **12**. The initial concentration of substrate was 1.0 mM. The PEC reactions were conducted at 0.15 V vs. Fc/Fc$^+$ for 1 h. An internal standard 1,3,5-trimethoxybenzene (4, 6.11 ppm, s, 3H) was added to quantify the epoxides (1, 3.98 ppm, s, 2H) in CD_3CN. The conversion of 4,4'-Br_2-*trans*-stilbene at 1 h was 89±10%, and the corresponding yield of epoxide **12** was 79±5%.

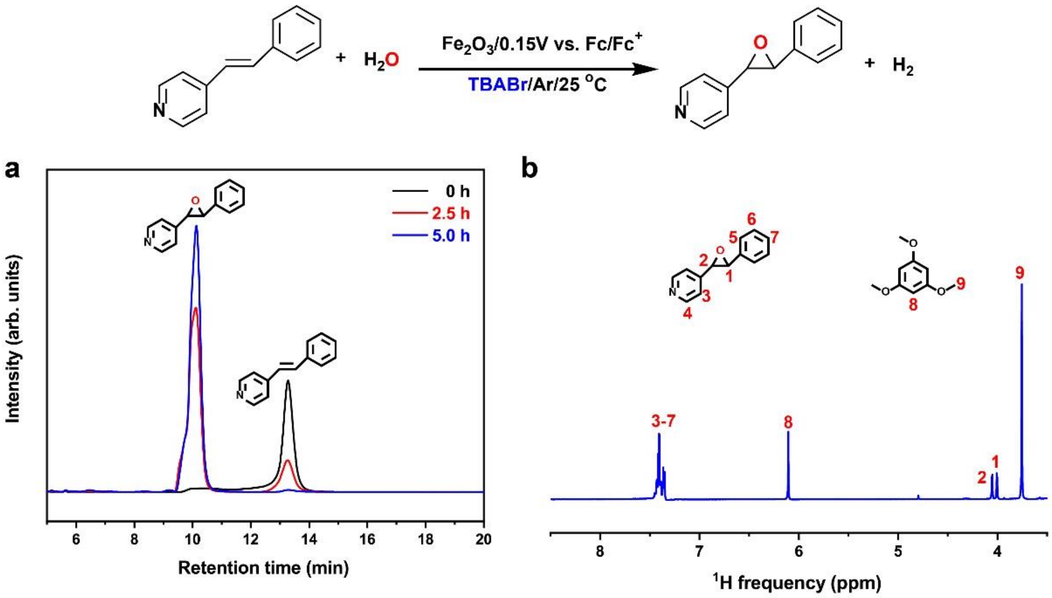

Supplementary Fig. 26 The HPLC spectra (**a**) and the corresponding ^{1}H NMR spectra (**b**) of **13**. The PEC reactions were conducted at 0.15 V vs. Fc/Fc$^+$ for 4 h. An internal standard 1,3,5-trimethoxybenzene (8, 6.11 ppm, s, 3H) was added to quantify the epoxides (1, 4.00 ppm, d, 1H; 2, 4.05 ppm, d, 1H) in CD_3CN. The conversion of 4-stilbazole at 5 h was 100%, and the corresponding yield of epoxide **13** was 96±2%.

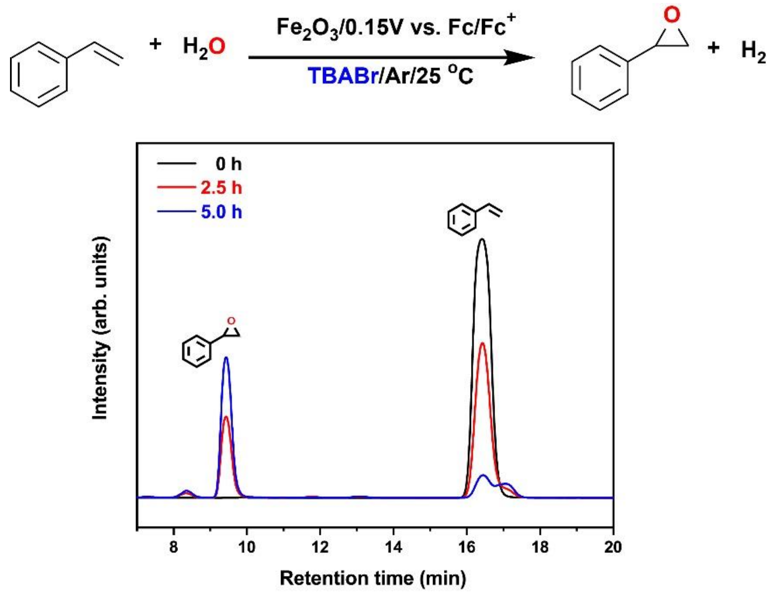

Supplementary Fig. 27 The HPLC spectra of **14**. The PEC reactions were conducted at 0.15 V vs. Fc/Fc^+ for 5 h. The substrate and product **14** were qualified by the standard curves. The conversion of styrene at 5 h was 84±3%, and the corresponding yield of epoxide **14** was 76±4%.

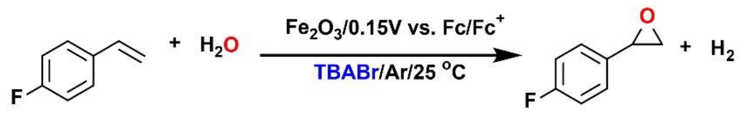

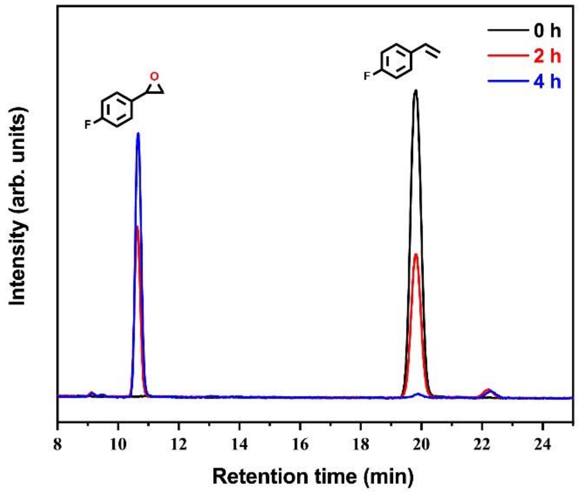

Supplementary Fig. 28 The HPLC spectra of **15**. The PEC reactions were conducted at 0.15 V vs. Fc/Fc$^+$ for 4 h. The substrate and product **15** were qualified by the standard curves. The conversion of 4-F-styrene at 4 h was 98±1%, and the corresponding yield of epoxide **15** was 95±3%.

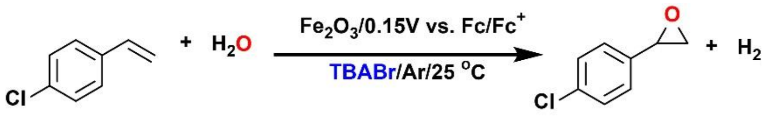

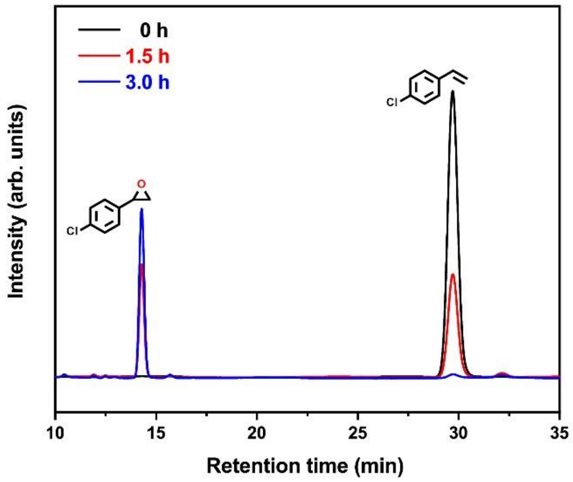

Supplementary Fig. 29 The HPLC spectra of **16**. The PEC reactions were conducted at 0.15 V vs. Fc/Fc^+ for 3 h. The substrate and product **16** were qualified by the standard curves. The conversion of 4-Cl-styrene at 3 h was 97±1%, and the corresponding yield of epoxide **16** was 88±4%.

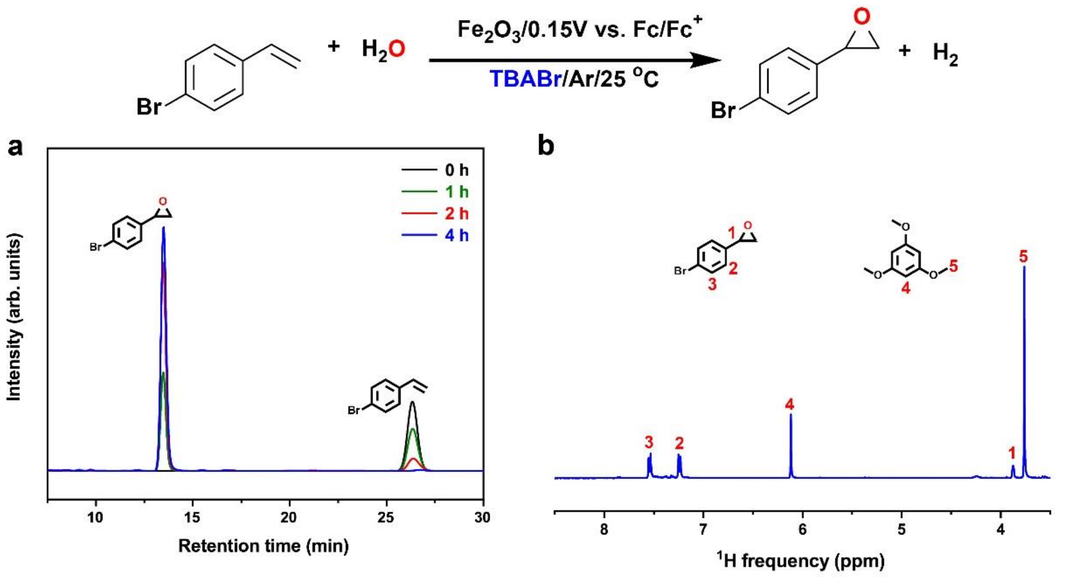

Supplementary Fig. 30 The HPLC spectra (**a**) and the corresponding ^{1}H NMR spectra (**b**) of **17**. The PEC reactions were conducted at 0.15 V vs. Fc/Fc^{+} applied bias for 4 h. An internal standard 1,3,5-trimethoxybenzene (4, 6.11 ppm, s, 3H) was added to quantify the epoxides (1, 3.88 ppm, t, 1H) in CD_3CN. The conversion of 4-Br-styrene at 4 h was 97±3%, and the corresponding yield of epoxide **17** was 87±6%.

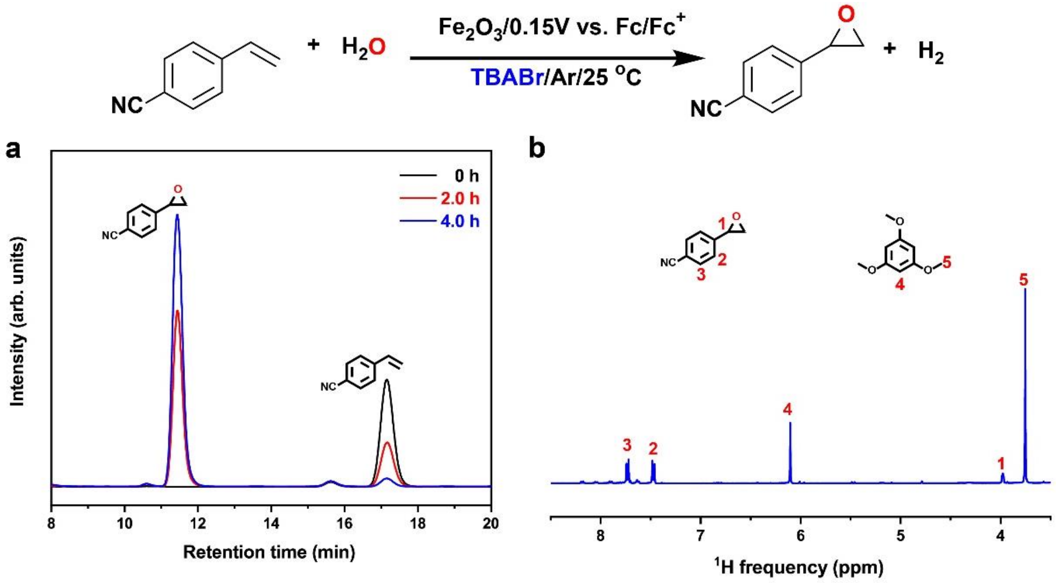

Supplementary Fig. 31 The HPLC spectra (**a**) and the corresponding ^{1}H NMR spectra (**b**) of **18**. The PEC reactions were conducted at 0.15 V vs. Fc/Fc^{+} for 4 h. An internal standard 1,3,5-trimethoxybenzene (4, 6.11 ppm, s, 3H) was added to quantify the epoxides (1, 3.98 ppm, t, 1H) in CD_3CN. The conversion of 4-CN-styrene at 4 h was 92±3%, and the corresponding yield of epoxide **18** was 82±7%.

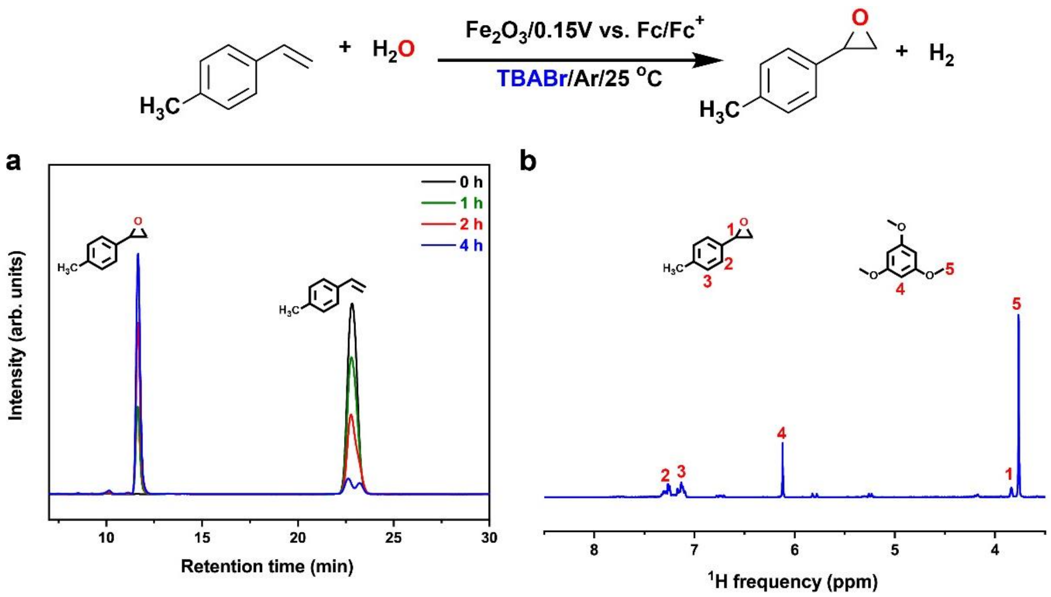

Supplementary Fig. 32 The HPLC spectra (**a**) and the corresponding ^{1}H NMR spectra (**b**) of **19**. The PEC reactions were conducted at 0.15 V vs. Fc/Fc$^+$ for 4 h. An internal standard 1,3,5-trimethoxybenzene (4, 6.11 ppm, s, 3H) was added to quantify the epoxides (1, 3.84 ppm, t, 1H) in CD_3CN. The conversion of 4-CH_3-styrene at 4 h was 85±5%, and the corresponding yield of epoxide **19** was 77±7%.

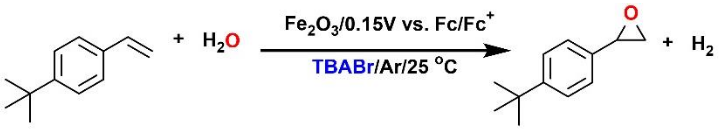

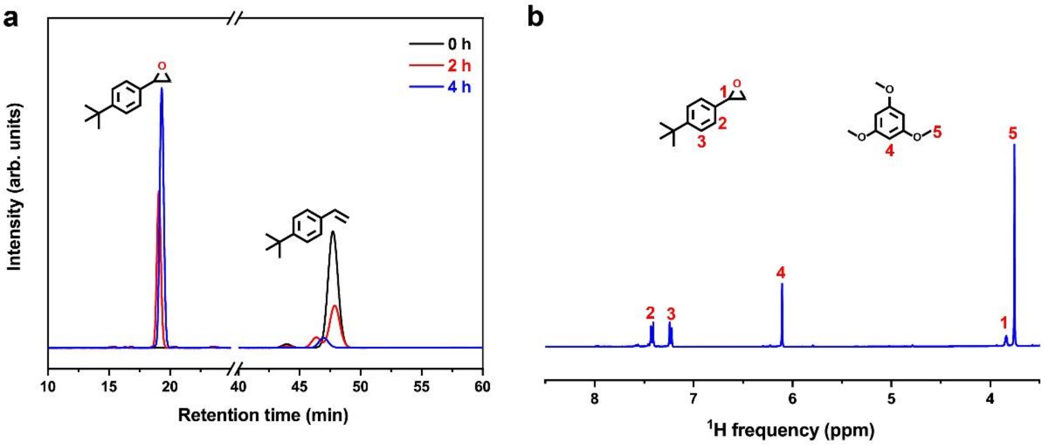

Supplementary Fig. 33 The HPLC spectra (**a**) and the corresponding ^{1}H NMR spectra (**b**) of **20**. The PEC reactions were conducted at 0.15 V vs. Fc/Fc^{+} for 4 h. An internal standard 1,3,5-trimethoxybenzene (4, 6.11 ppm, s, 3H) was added to quantify the epoxides (1, 3.84 ppm, t, 1H) in CD_3CN. The conversion of 4-tBu-styrene at 4 h was 92%, and the corresponding yield of epoxide **20** was 90±1%.

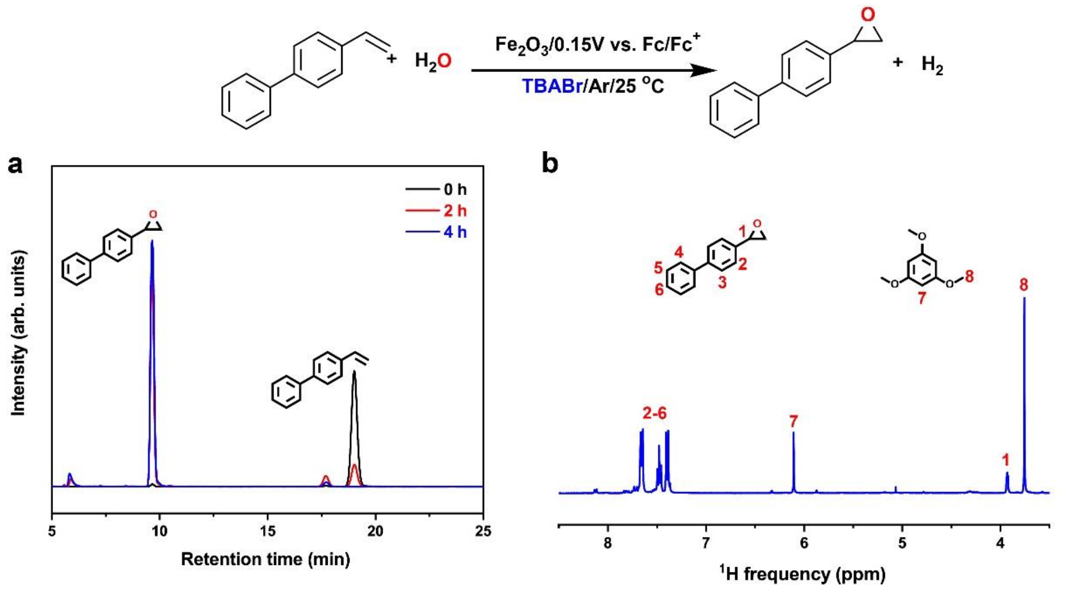

Supplementary Fig. 34 The HPLC spectra (**a**) and the corresponding ^{1}H NMR spectra (**b**) of **21**. The PEC reactions were conducted at 0.15 V vs. Fc/Fc^+ for 4 h. An internal standard 1,3,5-trimethoxybenzene (7, 6.11 ppm, s, 3H) was added to quantify the epoxides (1, 3.93 ppm, t, 1H) in CD_3CN. The conversion of 4-Ph-styrene at 4 h was 100%, and the corresponding yield of epoxide **21** was 98±1%.

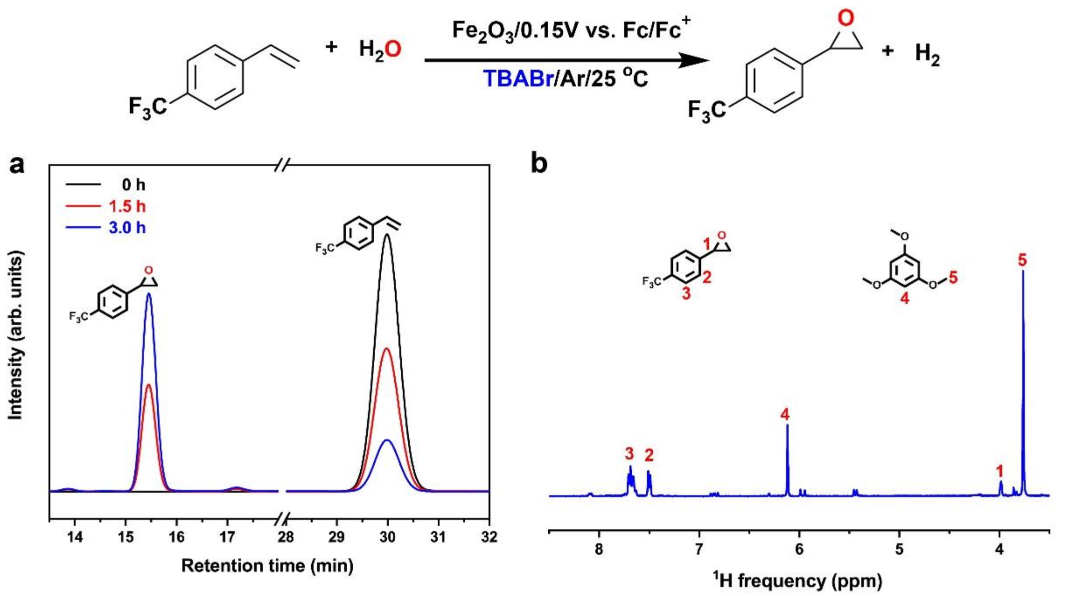

Supplementary Fig. 35 The HPLC spectra (**a**) and the corresponding ^1H NMR spectra (**b**) of **22**. The PEC reactions were conducted at 0.15 V vs. Fc/Fc^+ for 3 h. An internal standard 1,3,5-trimethoxybenzene (4, 6.11 ppm, s, 3H) was added to quantify the epoxides (1, 3.98 ppm, t, 1H) in CD_3CN. The conversion of 4-CF_3-styrene at 3 h was 78±4%, and the corresponding yield of epoxide **22** was 66±5%.

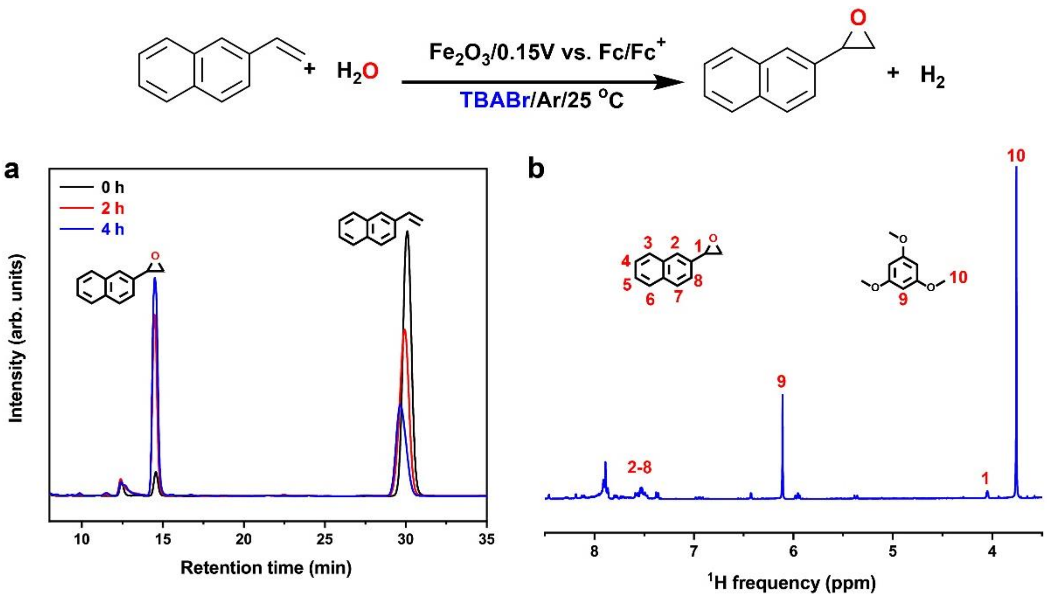

Supplementary Fig. 36 The HPLC spectra (**a**) and the corresponding ^{1}H NMR spectra (**b**) of **23**. The PEC reactions were conducted at 0.15 V vs. Fc/Fc^{+} applied bias for 4 h. An internal standard 1,3,5-trimethoxybenzene (9, 6.11 ppm, s, 3H) was added to quantify the epoxides (1, 4.05 ppm, t, 1H) in CD_3CN. The conversion of 2-Vinylnaphthalene at 4 h was 59±9%, and the corresponding yield of epoxide **23** was 40±1%.

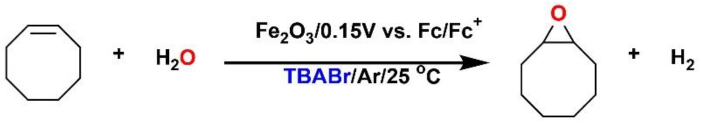

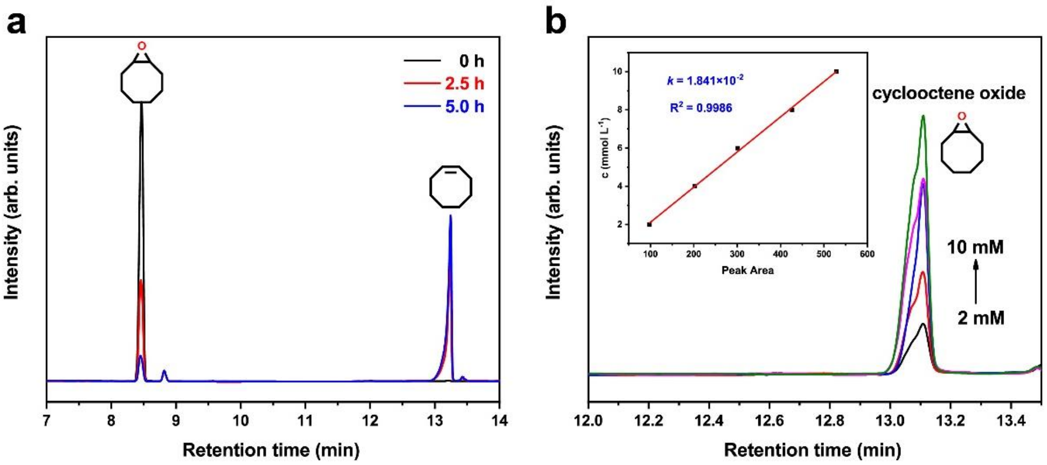

Supplementary Fig. 37 The gas chromatography (GC) spectra of (**a**) **24** and (**b**) standard cyclooctene oxide with different concentrations in 0.1 M TBABr acetonitrile and water (5 vol%) mixture solution. The PEC reactions were conducted at 0.15 V vs. Fc/Fc^+ applied bias for 5 h. The substrate and product **24** were qualified by the standard curves. The conversion of cyclooctene at 5 h was 92±1%, and the corresponding yield of epoxide **24** was 71±2%. The widening of GC peaks is also observed at all sample concentrations, and good linearity between the peak area and sample concentration is obtained, confirming that the broad peak only represents one product rather than two poorly separated products. Such a widening of GC peak may stem from the high polarity of product oxide.

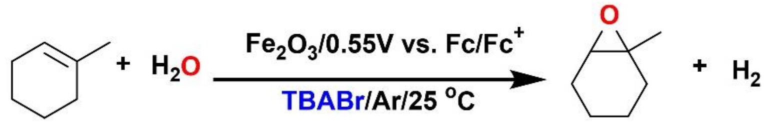

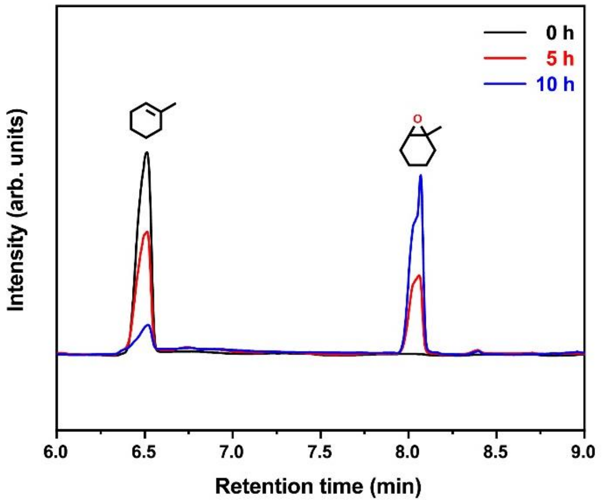

Supplementary Fig. 38 The GC spectra of **25**. The PEC reactions were conducted at 0.55 V vs. Fc/Fc^+ applied bias for 10 h. The substrate and product **25** were qualified by the standard curves. The conversion of 1-CH_3-cyclohexene at 10 h was 89±4%, and the corresponding yield of epoxide **25** was 87±5%.

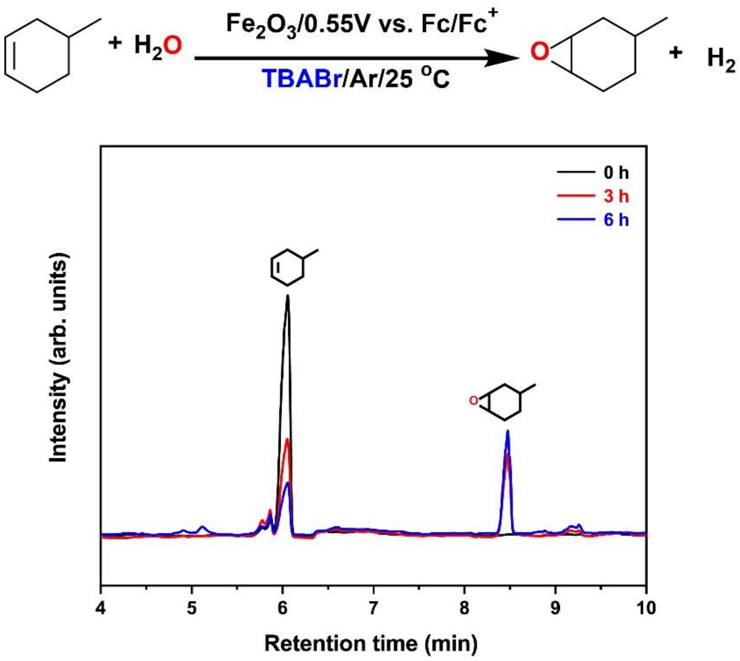

Supplementary Fig. 39 The GC spectra of **26**. The PEC reactions were conducted at 0.55 V vs. Fc/Fc^+ applied bias for 6 h. The substrate and product **26** were qualified by the standard curves. The conversion of 4-CH_3-cyclohexene at 6 h was 68±12%, and the corresponding yield of epoxide **26** was 42±5%.

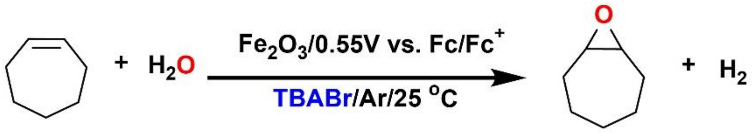

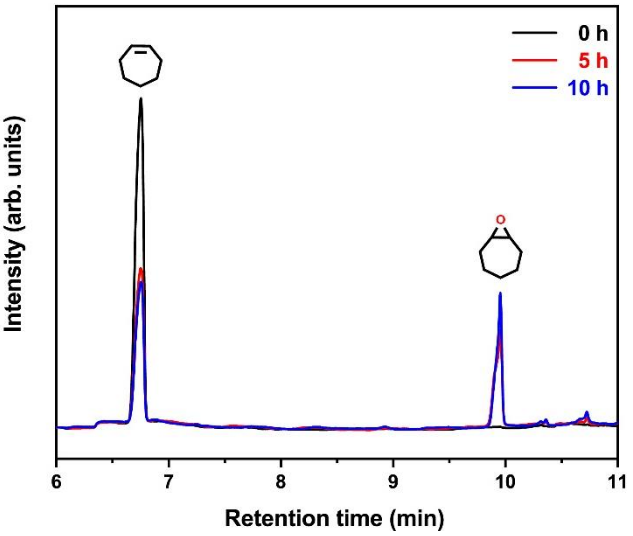

Supplementary Fig. 40 The GC spectra of **27**. The PEC reactions were conducted at 0.55 V vs. Fc/Fc$^+$ applied bias for 10 h. The substrate and product **27** were qualified by the standard curves. The conversion of cycloheptene at 10 h was 54±6%, and the corresponding yield of epoxide **27** was 29±5%.

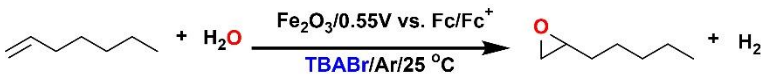

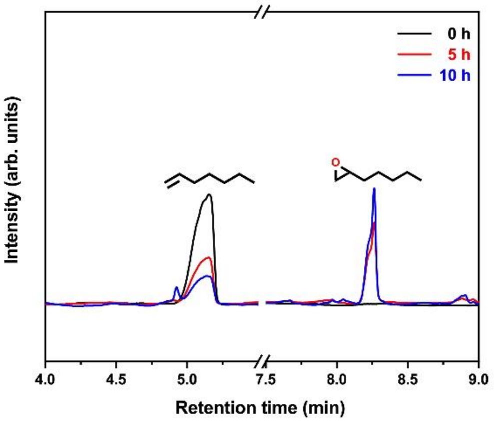

Supplementary Fig. 41 The GC spectra of **28**. The PEC reactions were conducted at 0.55 V vs. Fc/Fc$^+$ applied bias for 10 h. The substrate and product **28** were qualified by the standard curves. The conversion of 1-heptene at 10 h was 74±6%, and the corresponding yield of epoxide **28** was 47±2%.

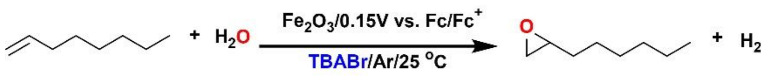

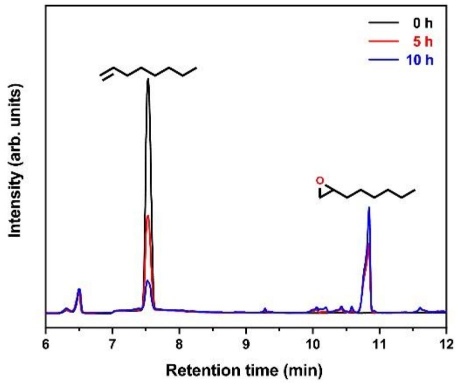

Supplementary Fig. 42 The GC spectra of **29**. The PEC reactions were conducted at 0.15 V vs. Fc/Fc^+ applied bias for 10 h. The substrate and product **29** were qualified by the standard curves. The conversion of 1-octene at 10 h was 83±5%, and the corresponding yield of epoxide **29** was 35±2%.

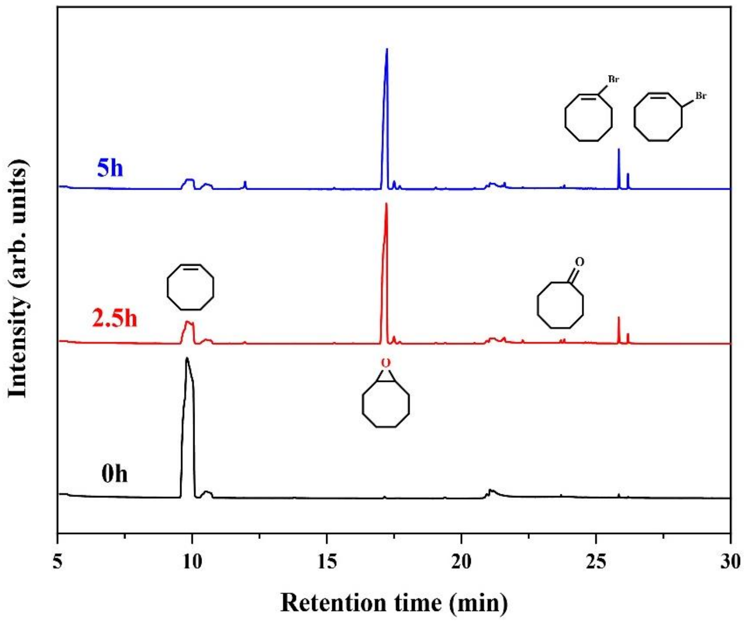

Supplementary Fig. 43 The GC spectra obtained at different PEC oxidation time of 10 mM cyclooctene with 100 mM TBABr at 0.15 V vs. Fc/Fc^{+} on α-Fe_2O_3. The products were analyzed by GC-MS data. In our systems, several by-products, including ketones, bromine-substituted products (as shown by GC-MS analysis) are detected.

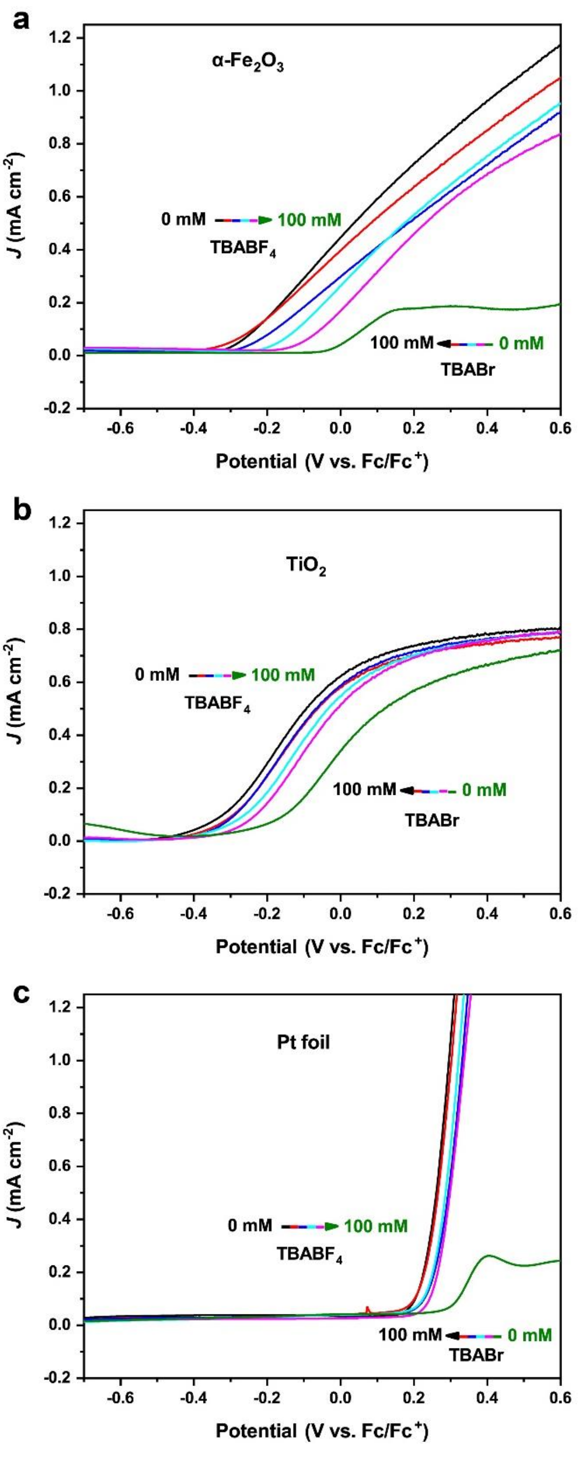

Supplementary Fig. 44 Linear sweep voltammetry curves on α-Fe_2O_3 (**a**) and TiO_2 (**b**) photoanodes under AM 1.5 G illumination, and Pt foil electrode (**c**) with different ratios of TBABr and $TBABF_4$ (total concentration of TBA^+: 100 mM, the concentration of Br^-: 100 mM, black; 75 mM, red; 50 mM, blue; 25 mM, cyan; 10 mM magenta; 0 mM, olive-green) as electrolyte; scan rate: 50 mV/s. In CH_3CN systems with 5% H_2O.

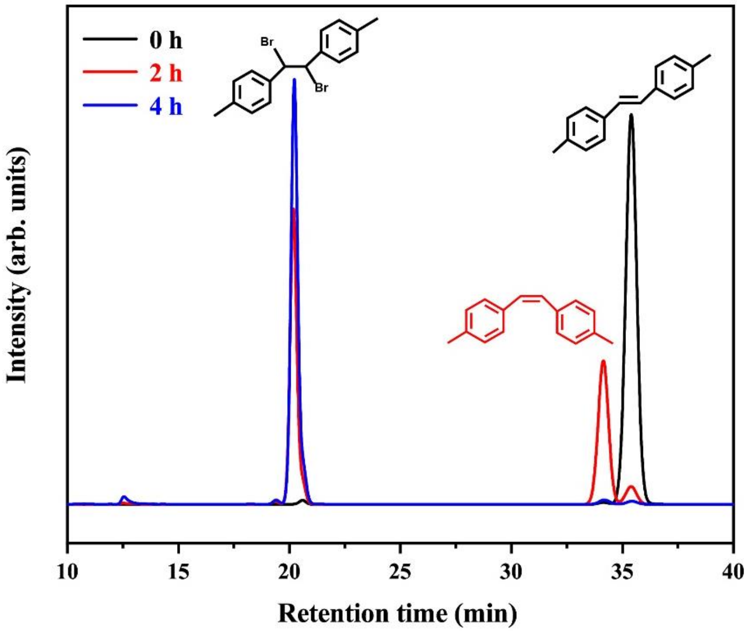

Supplementary Fig. 45 The HPLC spectra of substrate **1** with $[Ru(bpy)_3]Cl_2$-$Na_2S_2O_8$ under AM 1.5G illumination.

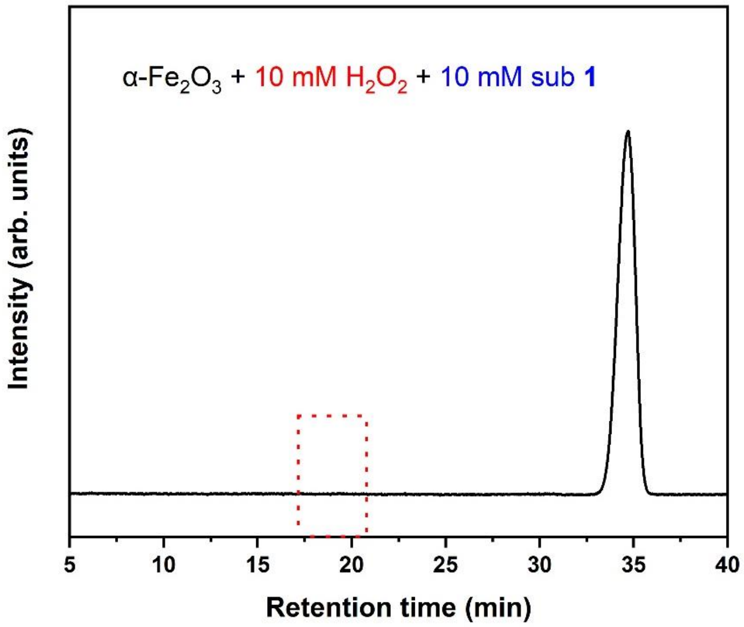

Supplementary Fig. 46 The HPLC spectra obtained after 4h' reaction time of 10 mM **1** and 10 mM H_2O_2 in the dark condition on α-Fe_2O_3. No epoxidation was detected in the presence of 10 mM H_2O_2 on α-Fe_2O_3, indicating that H_2O_2, if geneated by water oxidation, cannot be the dominant oxidant for the epoxidation of alkene in our Br-mediated system.

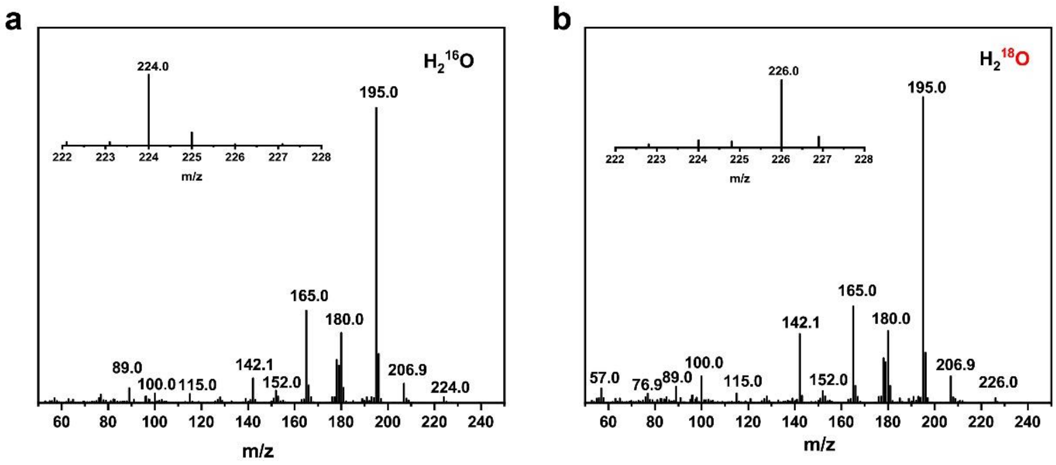

Supplementary Fig. 47 Mass spectra of product **2** during the oxidation of substrate **1** in the presence of $H_2{}^{16}O$ (**a**) or $H_2{}^{18}O$ (**b**).

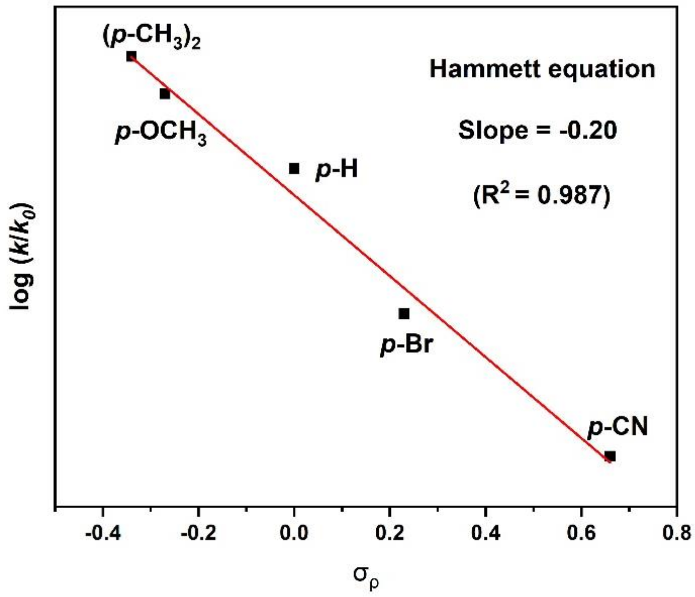

Supplementary Fig. 48 The Hammett plot with respect to σ_ρ values for the PEC epoxidation with various para-substituent *Trans*-stilbene substrates. The slope was calculated according to the Hammett equation: log $(k/k_0) = \rho \times \sigma_\rho$.

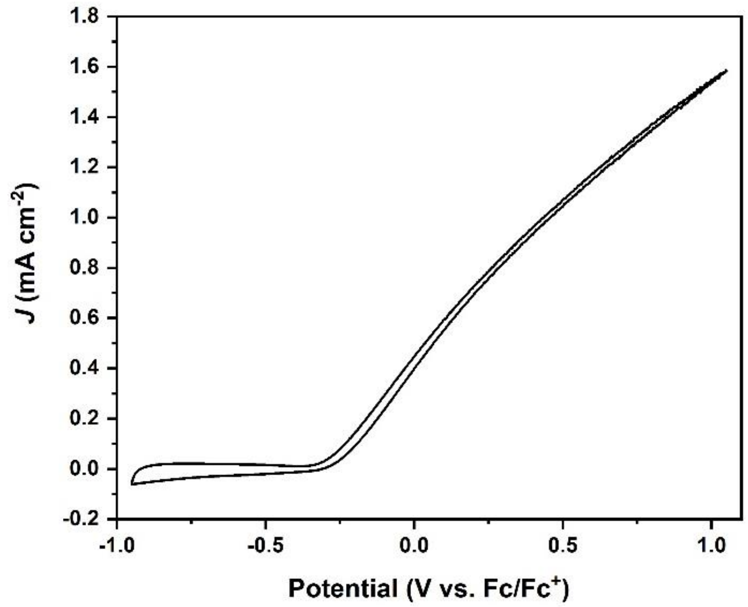

Supplementary Fig. 49 The CV curve of α-Fe_2O_3 measured in 0.1 M TBABr solution (CH_3CN with 5% H_2O) with 10 mM substrate **1** in an Ar atmosphere under AM 1.5G illumination.

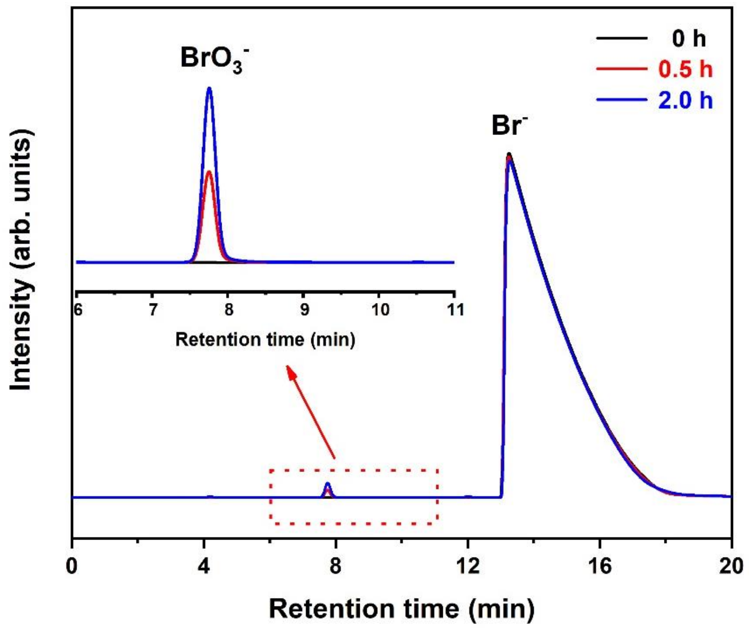

Supplementary Fig. 50 The IC spectra of reaction solution after 2 hours' photoelectrolysis. Some BrO_3^- ions were detected, which accounts for a FE of 13%.

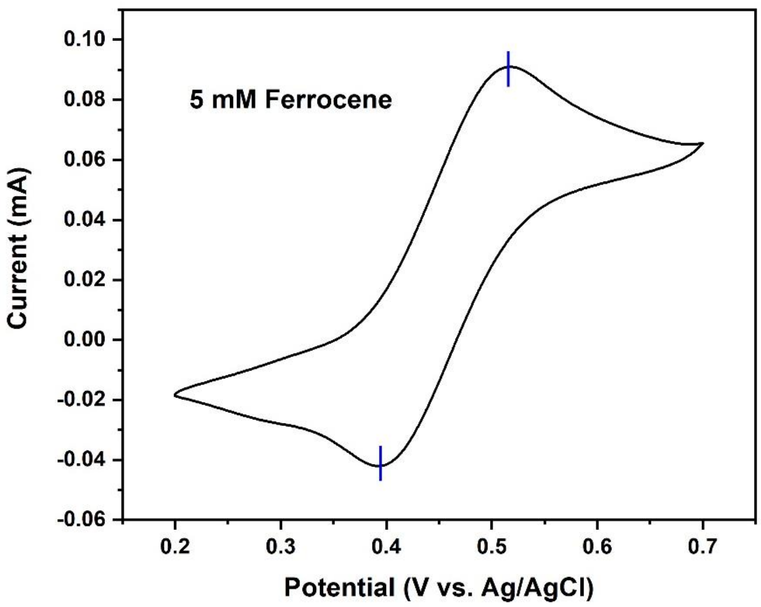

Supplementary Fig. 51 Cyclic voltammetry curves of 5.0 mM ferrocene solution by a glassy carbon electrode in CH_3CN with 5% H_2O in the presence of 0.1 M TBABr electrolyte (scan rate: 0.05 V s^{-1}). $E_{1/2}$ = 0.45 V vs. Fc/Fc^+.

Accordingly, the applied potential was calibrated according to the following equation: E(vs. Fc/Fc^+) = E(vs. Ag/AgCl) - 0.45 V.

Supplementary Table 1. Examples with best performance for previously reported PEC or EC olefin epoxidation.

Entry	Substrate	anode	mediator	Select. (%)	FE (%)	Yield (%)	Ref.
1	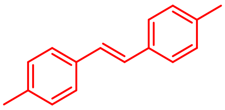	PEC on α-Fe_2O_3	TBABr	>99	82±4	97±1	This work
2	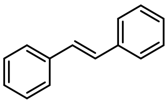	PEC on α-Fe_2O_3	no	72±2	12±1	59±2	[1][1]
3	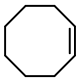	PEC on α-Fe_2O_3	no	9±1	3	8±1	[1][1]
4		EC on Pt foil	KCl	97	70	-	[2][5]
5	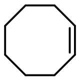	EC on Mn_3O_4	no	72	30	28	[3][6]
6	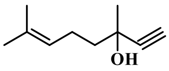	EC on Pt foil	NaBr	77	33	77	[4][7]
7	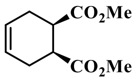	EC on Pt	NaBr	97	39	-	[5][8]
8	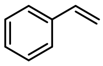	EC on Pt	NaBr	79	30	79	[6][9]
9	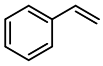	EC on Graphite	NaBr	83	40	80	[7][10]
10	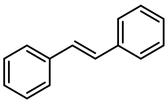	EC on CoS_2-CoS-GF	NaBr	99	14	40	[8][11]

Supplementary Table 2. The influence of TBABr percentage on selectivity, FE and yield for PEC epoxidation reactions on α-Fe_2O_3[a].

TBABr (%)	Selectivity (%)	FE (%)	Yield (%)[b]
0[c]	43±5	41±3	23±5
10	51	27	21
25	86	53	80
50	99	57	92
75	99	65	98
100	>99	82±4	97±1

[a]Reaction conditions: In CH_3CN systems with 5% H_2O, the total electrolyte concentration of TBA^+ was kept at 100 mM, and the initial concentration of substrate **1** was 10 mM; the PEC reaction was conducted at 0.15 V vs. Fc/Fc^+ to calculate the corresponding selectivity and FE of epoxide **2** for 2 h. [b]The yield of epoxide **2** was obtained at 4 h. [c]The PEC reaction was conducted at 0.75 V vs. Fc/Fc^+.

Supplementary Table 3. The influence of applied bias on the selectivity, FE and yield for PEC epoxidation reactions on α-Fe_2O_3[a].

Bias (V vs. Fc/Fc^+)	Selectivity (%)	FE (%)	Yield (%)[b]
0.15	>99	82±4	97±1
0.35	>99	77	97
0.55	97	70	92
0.75	98	67	98
1.05	94	53	93

[a]Reaction conditions: In CH_3CN systems with 5% H_2O, 100 mM TBABr was used as the electrolyte and the initial concentration of substrate **1** was 10 mM; The PEC reactions were conducted with different applied biases to calculate the corresponding selectivity and FE of epoxide **2** for 2 h. [b]The yield of epoxide **2** was calculated at 4 h, in which the substrate was completely consumed.

Supplementary Table 4. The influence of pH values of added water on selectivity, yield and FE for PEC epoxidation reactions on α-Fe_2O_3[a].

pH (H_2O)[b]	Selectivity (%)	FE (%)	Yield (%)[c]
3.0	86±6	50±6	82±7
5.0	98±1	70±5	98±1
6.4	>99	82±4	97±1
7.0	92±4	60±4	85±7
9.0	96±3	64±9	92±5
11.0	96±2	66±2	93±7

[a]Reaction conditions: In CH_3CN systems with 5% H_2O, 100 mM TBABr was used as the electrolyte, and the initial concentration of substrate **1** was 10 mM; The PEC reactions were conducted at 0.15 V vs. Fc/Fc^+ to calculate the corresponding selectivity and FE of the epoxide **2** for 2 h. All the error bars are defined in the table (s.d.) together with a measure of the mean. [b]The pH values of added H_2O, adjusted by HBr and TBAOH. [c]The yield of epoxide **2** was calculated at 4 h, in which the substrate was almost consumed.

In our system, the PEC reaction was carried out in CH_3CN solution with typically only 5 vol% H_2O as oxygen source. Considering that the term "pH" is not applicable in the non-aqueous solution, the pH value listed in Supplementary Table 4 refers to the pH of the added water to reflect relative acidity/basicity of solution, but not the pH of the whole CH_3CN solution. Under our experimental conditions, the stability of hypobromite and its pH-dependency, which may be largely different from those in the aqueous solution, is unclear. Nevertheless, the systematic comparisons on the product distribution of alkene oxidation by different Br active species (Br_2, Br· and BrO^-) under our solution conditions (Figs. 3d-3e and Supplementary Fig. 45), suggest that BrO^- is the most possible active species for the epoxidation reaction.

Supplementary Table 5. Scope of the aliphatic alkenes for the PEC bromine-mediated epoxidation[a].

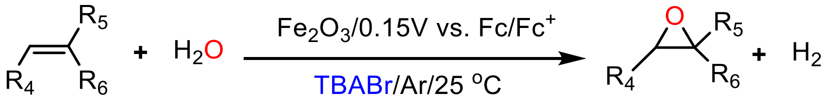

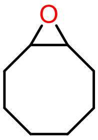	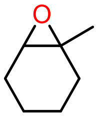	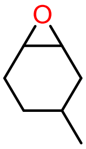	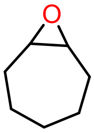
**24**[b] 75±4%(41±1%)	**25**[c] 87±11%(9±2%)	**26**[d] 62±3%(9±2%)	**27**[c] 48±7%(4±1%)
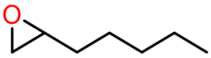	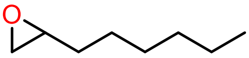		
**28**[c] 60±1%(6±1%)	**29**[c] 46±3%(7±1%)		

[a]Reaction conditions: substrates (10 mM) in CH_3CN (5% H_2O) in Ar atmosphere at ambient temperature at 0.15 V vs. Fc/Fc^+ applied bias. Selectivity and FE values (in brackets) were determined by GC (Supplementary Figs. 37-42). All the error bars are defined in the table (s.d., n=3 independent experiments) together with a measure of the mean. [b]2.5 h of reaction. [c]5.0 h of reaction. [d]3.0 h of reaction.

For the epoxidation of aliphatic alkenes, most of substrates behaved good selectivity, but the FE values are very poor except cyclooctene (FE, 41±1%). The relatively mediocre selectivity and FE values of aliphatic alkenes may be result from multiple factors (such as ring contraction reaction, side-reaction, self-disproportionate).

Supplementary References

1. Y. Zhao, C. Deng, D. Tang, L. Ding, Y. Zhang, H. Sheng, H. Ji, W. Song, W. Ma, C. Chen, J. Zhao, α-Fe_2O_3 as a versatile and efficient oxygen atom transfer catalyst in combination with H_2O as the oxygen source. *Nat. Catal.* **4**, 684-691 (2021).
2. N. T. Hahn, C. B. Mullins, Photoelectrochemical Performance of Nanostructured Ti- and Sn-Doped α-Fe_2O_3 Photoanodes. *Chem. Mater.* **22**, 6474-6482 (2010).
3. J. Huang, G. Hu, Y. Ding, M. Pang, B. Ma, Mn-doping and NiFe layered double hydroxide coating: Effective approaches to enhancing the performance of α-Fe_2O_3 in photoelectrochemical water oxidation. *J. Catal.* **340**, 261-269 (2016).
4. Y. Zhao, T. Shi, J. Shang, L. Ding, X. Cao, C. Chen, J. Zhao, Rapid proton exchange between surface bridging hydroxyls and adsorbed molecules on TiO_2. *Appl. Catal. B* **277**, (2020).
5. W. R. Leow, Y. Lum, A. Ozden, Y. Wang, D.-H. Nam, B. Chen, J. Wicks, T.-T. Zhuang, F. Li, D. Sinton, E. H. Sargent, Chloride-mediated selective electrosynthesis of ethylene and propylene oxides at high current density. *Science* **368**, 1228–1233 (2020).
6. K. Jin, J. H. Maalouf, N. Lazouski, N. Corbin, D. Yang, K. Manthiram, Epoxidation of Cyclooctene Using Water as the Oxygen Atom Source at Manganese Oxide Electrocatalysts. *J. Am. Chem. Soc.* **141**, 6413-6418 (2019).
7. S. Torii, K. Uneyama, M. Ono, H. Tazawa, S. Matsunami, A regioselective ω-epoxidation of polyisoprenoids by the sodium bromide promoted electrochemical oxidation. *Tetraghedron Lett.* **48**, 4461-4462 (1979).
8. S. Torii, K. Uneyama, M. Ono, H. Tazawa, S. Matsunami, Efficient Conversion of Olefins into Epoxides, Bromohydrins, and Dibromides with Sodium Bromide in Water-Organic Solvent Electrolysis. *J. Org. Chem.* **46**, 3312-3315 (1981).
9. M. Inês, A. J. Mendonça, A. P. Esteves, D. I. Mendonça, M. J. Medeiros, Electroepoxidation of natural and synthetic alkenes mediated by sodium bromide. *C. R. Chimie* **12**, 841-849 (2009).
10. W. Jud, C. O. Kappe, D. Cantillo, One - pot multistep electrochemical strategy for the modular synthesis of epoxides, glycols, and aldehydes from alkenes. *Electrochem. Sci. Adv.* **1**, e2100002 (2021).
11. Y. Zhang, A. Iqbal, J. Zai, S.-Y. Zhang, H. Guo, X. Liu, I. ul Islam, H. Fazal, X. Qian, Bromine and oxygen redox species mediated highly selective electro-epoxidation of styrene. *Org. Chem. Front.* **9**, 436-444 (2022).
